# The hypoxic niche enclosing the shoot apical meristem is shaped by a combination of morphological features and metabolic activity

**DOI:** 10.1016/j.molp.2026.02.011

**Published:** 2026-05-04

**Authors:** Viktoriia Voloboeva, Bart Dequeker, Leen Van Doorselaer, Gabriele Panicucci, Pierdomenico Perata, Pieter Verboven, Bart Nicolai, Daan A. Weits

**Affiliations:** 1 Experimental and Computational Plant Development, Institute of Environment Biology, Utrecht University, Padualaan 8, 3584 CH Utrecht, the Netherlands; 2 PlantLab, Institute of Plant Sciences, Scuola Superiore Sant'Anna, 56010 Pisa, Italy; 3 National Enterprise for NanoScience and Nanotechnology, 56010 Pisa, Italy; 4 Division of Mechatronics, Biostatistics and Sensors (MeBioS), Department of Biosystems, KU Leuven, Willem de Croylaan 42, 3001 Leuven, Belgium; 5 Flanders Centre of Postharvest Technology, Willem de Croylaan 42, 3001 Leuven, Belgium

**Keywords:** shoot apical meristem, hypoxia, tissue compactness, cuticle, respiration

## Abstract

Stem cell niches in both plants and animals are frequently located in low-oxygen microenvironments that support their function. In plants, these hypoxic niches promote local stabilization of several transcriptional regulators that control a range of developmental processes, including shoot apical meristem (SAM) activity, vernalization, lateral root development, and leaf growth and morphogenesis. Despite their importance, however, it remained unclear how these hypoxic niches are maintained. In this study, we employed a combination of experimental and modeling approaches to identify the key features required to establish and sustain the hypoxic niche enclosing the SAM. Using respiration inhibitors, manipulation of resource availability, and mitochondria mutant lines, we found that respiratory oxygen consumption is required to establish the hypoxic niche. Oxygen microprofiling and imaging of hypoxia signaling in cuticle biosynthesis mutants, as well as following targeted cuticle degradation, revealed that a cuticle-like barrier defines the steepness of the oxygen gradient and ensures that even the outermost layer remains hypoxic. Moreover, high tissue compactness in the shoot apex region was visualized using X-ray micro-computed tomography and shown to stabilize the hypoxic microenvironment by limiting internal oxygen diffusion. Finally, sensitivity tests on a novel reaction-diffusion model closely recapitulated oxygen gradients across the SAM and revealed distinct roles of each feature and their combined effect on oxygen distribution. Together, these findings explain how the SAM sustains hypoxia and point to a potential universal strategy used by stem cell niches to maintain low oxygen levels.

## Teaser

This study reveals how a functional hypoxic microenvironment enclosing the shoot apical meristem arises through respiratory oxygen consumption and is stabilized by a cuticle-like diffusion barrier and dense cell organization. Our findings clarify how chronic hypoxia, essential for maintaining meristem activity, is sustained in plants and offer new strategies to manipulate oxygen status to influence meristem activity, developmental timing, and plant architecture.

## Introduction

Endogenous low oxygen levels occur widely in plant and animal bodies throughout their development and growth. These chronically hypoxic zones are essential for maintaining stem cell functions but also hamper efficient energy conversion. In animals, mesenchymal, neural, and hematopoietic stem cell niches typically maintain 1- to 9-kPa oxygen to preserve stem cell identity and pluripotency (Ezashi et al., 2005; Mohyeldin et al., 2010; Di Mattia et al., 2021). In plants, low-oxygen conditions are found in tissues that lack photosynthetic activity, are bulky, enclosed by gas barriers, or undergo active cell proliferation (van Dongen and Licausi, 2015; Weits et al., 2019). For instance, organs such as fruit, tubers, seeds, nodules, anthers, and roots contain oxygen levels below 5 kPa (Borisjuk and Rolletschek, 2009; Licausi et al., 2011; van Dongen and Licausi, 2015; Xiao et al., 2024). Hypoxia in seeds supports dormancy and oxidative protection, while nodules maintain low oxygen via leghemoglobin and suberin barriers to enable nitrogen fixation (De Giorgi et al., 2015; Venado et al., 2022). Localized hypoxia in anther tissue was shown to set germ cell fate (Kelliher and Walbot, 2012), it also promotes lateral root initiation during primordia formation (Shukla et al., 2019), and the shoot apical meristem (SAM) maintains a hypoxic microenvironment important for proper leaf initiation, cell expansion and the transition to flowering (Gibbs et al., 2018; Weits et al., 2019; Osborne et al., 2025). Recently, cyclic fluctuations in oxygen levels were revealed to regulate leaf growth (Triozzi et al., 2024). In addition, progressive oxygenation from the tip toward the leaf base was discovered to regulate leaf morphogenesis, further expanding the role for internal hypoxia in developmental processes (Panicucci et al., 2025). Hence, in these tissues, hypoxia is not just an incidental feature of tissue structure and metabolic activity but is likely also a functionally significant state that has been co-opted for developmental and physiological processes.

Meristematic niches are unique in that, despite direct exposure to atmospheric oxygen levels and lack of insulating bulky tissue, they maintain hypoxia within their small and localized domains. Indeed, remarkable oxygen gradients from ambient air (21.3 kPa O_2_) to the SAM (3–5 kPa O_2_) occur over a distance of only 10–20 μm (Weits et al., 2019). Despite these unique features and the importance of hypoxia in developmental processes, it still remains unknown how such steep oxygen gradients are established at meristems. In animals, hypoxia in stem cell niches is often due to restricted blood supply in areas distant from blood vessels (Parmar et al., 2007). Instead, plants lack an active oxygen-transport system, relying on diffusion driven by concentration gradients (Burg and Burg, 1965). Hence, oxygen levels in plant tissue are a result of oxygen diffusion resistance and respiratory consumption. Plants can also produce their own oxygen via photosynthesis, but the SAM is commonly described as a non-photosynthetically active region with undifferentiated proplastids (Lopez-Juez and Pyke, 2005). Hence, the SAM likely relies solely on oxygen diffusion from the environment and its surrounding tissue. Bearing this in mind, we previously proposed three hypotheses that inspired our investigation into meristematic oxygen gradients (Weits et al., 2021).

The first proposes the presence of an oxygen diffusion barrier covering the meristem. In this study, we focused on the cuticle, a protective layer on the outer surface of the epidermis composed of a hydrophobic biopolyester (cutin) and waxes that restricts gas diffusion in various plant tissues. For example, the cuticle layer on endosperm cells maintains hypoxia in seeds (De Giorgi et al., 2015). Amphibious plants modify cuticle thickness and composition in leaves to optimize gas exchange in aerial or submerged environments (Frost-Christensen et al., 2003). Indeed, studies show that the *Lycopersicon* cuticle has much lower permeability (1.1 × 10^−6^ m/s) than an equally thick water layer (∼2.4 × 10^−3^ m/s) (Lendzian, 1982). Given that the SAM is not embedded in bulky tissue and the cuticle is its only barrier to the environment, we investigated whether the cuticle plays a role in restricting oxygen diffusion. Secondly, it has also been proposed that high metabolic activity coupled to high oxygen consumption throughout the SAM tissue is a primary driver of hypoxia in hypoxic niches (Le Gac and Laux, 2019). Thirdly, we considered restricted gas diffusion caused by tight cell clustering, which limits oxygen diffusion and has previously been shown to play a major role in gas exchange in fruit and leaves (Retta et al., 2024; Xiao et al., 2024). In all scenarios, oxygen consumption exceeds supply.

Here, we first experimentally tested the three aforementioned features: a cuticle gas barrier, respiratory oxygen consumption, and tissue compactness. We then combined experimental data with a SAM reaction-diffusion model of oxygen and found that their combined effects establish and maintain the hypoxic niche of the SAM, with distinct functions for each factor. We determined that removing the cuticle barrier alters the steepness of the oxygen gradient from outside to the SAM and that this affects meristem size. We showed that mitochondrial respiration contributes to overall SAM oxygen consumption, tested how resource availability affects respiratory activity of SAMs, and revealed that actively dividing SAMs are more hypoxic than quiescent ones. Tissue compactness in the SAM and adjacent tissues was also visualized across developmental stages and correlated with hypoxic conditions. Finally, we tested the sensitivity of a simulated meristem model to changes in cuticle permeability, oxygen diffusivity, and respiration rate and compared the results with available experimental data. Together, our findings identify three key features that maintain the hypoxic microenvironment in the SAM and show how their modification affects endogenous oxygen status. This sheds light on how oxygen dynamics within the SAM influence plant development and may inform strategies to modify plant architecture by manipulating internal oxygen levels. Furthermore, our findings provide a foundation for exploring other hypoxic niches, aiding in their identification and characterization, and potentially predicting their formation in previously unstudied tissues or plant species.

## Results

### Spatial oxygen gradients in shoot meristems reflect tissue density and morphological organization

**Figure 1:**
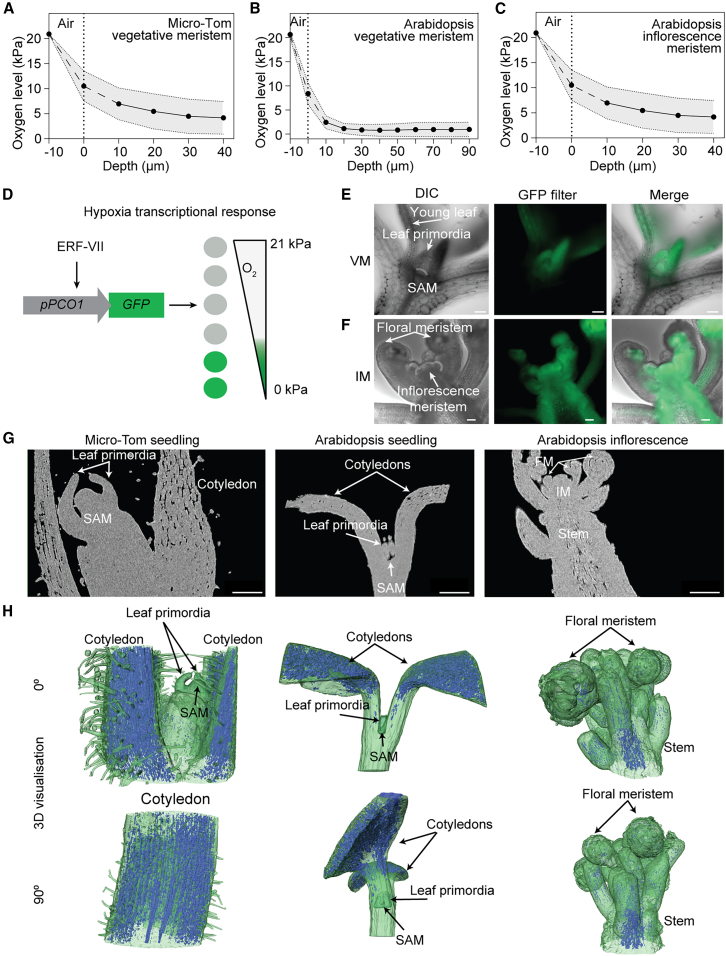
Oxygen measurements and tissue compactness in the SAMs. **(A–C)** Tomato seedling (*n* = 5), *Arabidopsis* 4-week-old plant (*n* = 3), and *Arabidopsis* inflorescence (*n* = 5) performed using a Clark-type oxygen microsensor with a 3- to 10-μm tip. The vertical stippled line denotes the estimated position of the SAM boundary using microscopy. The dashed segment of the oxygen microprofile indicates the transition of the sensor across this boundary, where oxygen levels are less certain. **(D)** The output scheme of transcriptional-based hypoxia reporter *pPCO1:GFP-GUS* at different endogenous oxygen levels. Gray circles represent no signal. Work principle: under hypoxia, the stabilized ERF-VII transcription factor activates the *PCO1* promoter, which contains an ERF-VII-binding site. As a result, *PCO1* promoter activity increases upon ERF-VII stabilization and decreases in the presence of oxygen when ERF-VII is degraded (Weits et al., 2014). **(E and F)** Fluorescence GFP signal of *pPCO1:GFP-GUS* expression in the SAMs of *Arabidopsis* seedling and inflorescence (*n* = 5). Differential interference contrast (DIC) images represent the bright field for better contrast. Scale bar, 50 μm. **(G)** X-ray μCT images of an *Arabidopsis* seedling, Micro-Tom seedling, and *Arabidopsis* inflorescence (*n* = 3). Scale bar, 0.2 mm. **(H)** 3D representation of gas spaces in the *xz* and *yz* dimensions (*n* = 3) at two different rotation angles (0° and 90°) around the vertical axis. The blue color indicates the presence of gas spaces, while the green color shows compact tissue. The regions of the SAM, inflorescence meristem (IM), and flowering meristem (FM) are indicated.

To assess whether steep oxygen gradients occur across different developmental stages, we measured oxygen profiles in both vegetative and inflorescence SAMs using a Clark-type oxygen microsensor (Supplemental Figure 1). Consistent with previous findings (Weits et al., 2019), tomato seedling SAMs exhibited a pronounced oxygen gradient (Figure 1A). An even steeper decline was found in 4-week-old *Arabidopsis* SAMs, with oxygen levels approaching 1 kPa (Figure 1B). The *Arabidopsis* inflorescence meristem (IM) also displayed low oxygen and a steep gradient (Figure 1C). We then sought to identify common factors contributing to hypoxia in shoot meristems. A defining morphological feature of the SAM is its small size and dense arrangement of actively dividing cells, which may limit oxygen diffusion. To explore whether tissue compactness correlates with oxygen levels, we first visualized low-oxygen signaling in the SAM compared to surrounding tissue, which is challenging to achieve in 2D using physical sensors (Panicucci et al., 2024). We therefore took advantage of an existing transcription-based hypoxia reporter in which the hypoxia-inducible *PLANT CYSTEINE OXIDASE 1* (*PCO1*) promoter drives β-glucuronidase (GUS) and green fluorescent protein (GFP) reporter genes (Weits et al., 2014) (Figure 1D). We imaged vibratome longitudinally sectioned *Arabidopsis* seedling and adult plant inflorescences expressing *pPCO1:GUS-GFP*. In seedlings, GFP signal was detected in the meristem, leaf primordia, and young leaves, while the cotyledon petioles and hypocotyl showed no signal, indicating that the shoot apex is specifically enclosed in a hypoxic niche (Figure 1E). In the IM, the reporter was active in the SAM, floral primordia, and flanks of the stem but not in the central stem region (Figure 1F). To further validate hypoxia response distribution, we imaged a hypoxia reporter based on a five-time-repeat of a minimal *Hypoxia-Responsive Promoter Element* (*HRPE*), *pHRPEx5:GUS-GFP*, and a hybrid transcriptional and maturation-based biosensor, *pHRPEx5:UnaG-mCherry* (Panicucci et al., 2020). Both HRPEx5-type reporters revealed a similar pattern of hypoxia signaling at the vegetative SAM and IM (Supplemental Figure 2A–2C).

To test how low oxygen levels are correlated to tissue microstructure, we used X-ray micro-computed tomography (μCT) imaging to assess tissue compactness in *Arabidopsis* and tomato meristems and surrounding tissues. μCT provided contrast between the liquid and gas phases (Figure 1G), showing dense, nearly air-free meristem tissue in all SAM samples, compared to more porous cotyledons and stems (Figure 1G and 1H). In *Arabidopsis* seedlings, the region below the vegetative SAM also showed tightly packed cells, although this was not correlated with *pPCO1:GUS-GFP* or *HRPEx5* activation (Figure 1E, 1G, and 1H; Supplemental Figure 2). The stem below the *Arabidopsis* inflorescence showed densely packed cells at the periphery, but not in the center, correlating with *pPCO1:GUS-GFP* and *HRPEx5* signal (Figure 1F and 1H; Supplemental Figure 2).

To study the effect of more densely packed cells on internal SAM gas diffusion, we used a *clv3-15* knockout mutant, showing SAM enlargement and meristem bulging resulting in more leaves being produced (Supplemental Figure 3A and 3B) (Forner et al., 2015). Surprisingly, we found that oxygen levels in *clv3-15* were higher than in wild-type (WT) SAMs (Supplemental Figure 3C). However, loss-of-function *clv3-15* mutants have been described as having undefined zonal organization and a lower proportion of cells undergoing mitosis (Laufs et al., 1998; Rambaud-Lavigne et al., 2024). Hence, the pleiotropic effects on cell proliferation in this mutant likely confound any effect of meristem size on internal oxygen levels.

While the above findings revealed that tissue compactness and oxygen status correlate, it is not sufficient to explain SAM hypoxia, hinting at other important features that contribute to local hypoxia.

### A cuticle-like layer covers the SAM and acts as a gas barrier

**Figure 2:**
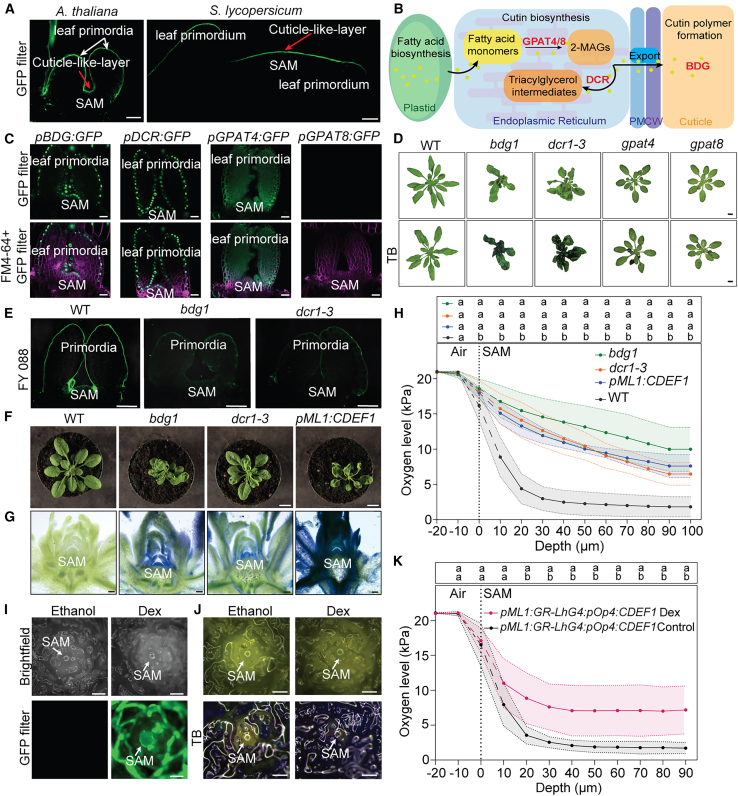
Cuticle defects and their effect on oxygen levels in the SAMs. **(A)** Confocal microscopy showing Fluorol Yellow 088 (FY) staining of the SAMs of a 4-day-old *Arabidopsis* seedling and 10-day-old tomato seedling. Scale bars, 20 μm (*n* = 4–6). **(B)** Schematic diagram of the cuticle biosynthetic pathway. Fatty acids formed in plastids are transferred to the endoplasmic reticulum for the synthesis of sn-2 monoacylglycerols (2MAG) by GLYCEROL-3-PHOSPHATE ACYLTRANSFERASEs (GPATs). 2MAG is acylated to triacylglycerol in the presence of DEFECTIVE IN CUTICULAR RIDGES (DCR). Triacylglycerol can be hydrolyzed by BODYGUARD (BDG) to generate hydroxy fatty acids that can be further polymerized. **(C)** Confocal microscopy images showing promoter activity in plants expressing *pBDG1:GFP*, *pDCR:GFP*, *pGPAT4:GFP*, and *pGPAT8:GFP* in the SAM of 4-day-old seedlings. FM4-64 staining was used to stain membranes, false color in magenta. Scale bars, 20 μm (*n* = 6–10). **(D)** TB staining indicating cuticle permeability in the *bdg1*, *dcr1-3*, *gpat4*, and *gpat8* compared to WT plants. The top images show the plants before staining and the bottom images after staining. Scale bar, 1 cm (*n* = 5). **(E)** Confocal microscopy images of the FY staining in the SAM of cuticle biosynthesis mutants *bdg1* and *dcr1-3* compared to WT, demonstrating cuticle defects. Imaging was performed using confocal laser scanning microscopy Zeiss Airyscan 800 (excitation, 488 nm; detection, 410–575 nm). Scale bar, 20 μm (*n* = 10–15). **(F)** Pictures of plant rosettes were taken with a Nikon Coolpix P520 digital camera. Scale bar, 1 cm (*n* = 6). **(G)** TB staining of SAM vibratome sections from *bdg1*, *dcr1-3*, and *pML1:CDEF1* 4-week-old plants showing blue dye infiltration inside the meristems in comparison to WT plants. Section thickness is 120 μm. Scale bar, 200 μm (*n* = 7–11). **(H)** Oxygen microprofiling in the SAMs of *bdg1*, *dcr1-3*, *pML1:CDEF1*, and WT plants performed using a Clark-type oxygen microsensor with a 10-μm tip. Oxygen levels were measured every 10 μm up to a depth of 100 μm. The vertical stippled line denotes the estimated position of the SAM boundary using microscopy. The dashed segment of the oxygen microprofile indicates the transition of the sensor across this boundary, where oxygen levels are less certain. Statistical differences were evaluated using two-way repeated-measures ANOVA, followed by Tukey’s multiple-comparisons test (at each depth); *p* < 0.05 (*n* = 6). **(I)** mTurquoise visualization in the SAM of 4-week-old *pML1:GR-LHG4:pOp4:CDEF1* plants treated with Dex or ethanol solutions. Scale bar, 2 mm (*n* = 8). **(J)** TB staining of SAMs from 4-week-old *pML1:GR-LHG4:pOp4:CDEF1* plants, showing blue dye penetration in the SAM after Dex treatment compared to ethanol-treated controls. Scale bar, 2 mm (*n* = 5). **(K)** Oxygen measurements in the SAMs of *pML1:GR-LHG4:pOp4:CDEF1* plants after Dex and ethanol treatments, measured with a Clark-type oxygen microsensor with a 10-μm tip. Oxygen levels were measured every 10 μm to a depth of 100 μm. The vertical stippled line denotes the estimated position of the SAM boundary using microscopy. The dashed segment of the oxygen microprofile indicates the transition of the sensor across this boundary, where oxygen levels are less certain. Statistical differences were evaluated using two-way repeated-measures ANOVA, followed by Šídák’s multiple-comparisons test (at each depth), *p* < 0.05 (*n* = 5–6).

Evidence from the literature showed that the cuticle can act as gas barrier under certain conditions (Frost-Christensen et al., 2003; De Giorgi et al., 2015; Venado et al., 2022). Using the Fluor Yellow 088 fluorescent dye, we identified a lipophilic structure in the shoot apex of *Arabidopsis thaliana* and *Solanum lycopersicum* that covers the meristem and leaf primordia (Figure 2A). To confirm whether this layer is related to the cuticle, we examined the expression pattern, using promoter:GFP reporters, of several genes involved in cutin leaf biosynthesis: *GLYCEROL-3- PHOSPHATE ACYLTRANSFERASEs* (*GPAT4*, *GPAT8*), *DEFECTIVE IN CUTICULAR RIDGES* (*DCR*), and *BODYGUARD* (*BDG1*) (Figure 2B; Berhin et al., 2019). The expression of *BDG1* and *DCR* was detected in the outermost layer of the meristem and primordia (Figure 2C). *GPAT4* expression was distributed throughout the meristematic area, suggesting that the activity of this gene is not specific to epidermal cells (Figure 2C). *GPAT8* expression was not detected in the SAM, suggesting that *GPAT8* expression is not required for SAM cuticle formation (Figure 2C).

Since we found that the SAM is covered by a cuticle, we reasoned that knockout lines of its biosynthetic genes could be used to determine the effect of the cuticle on low oxygen levels in the SAM. After identifying homozygous transfer DNA (T-DNA) insertion lines for *bdg1*, *gpat4*, *gpat8*, and *dcr1-3* (Supplemental Figure 4A–4D), we examined the phenotype of these mutants. The knockout of *BDG* and *DCR* were found to cause the fusion of leaves, characterizing cuticle alterations (Figure 2D) as shown before (Lolle et al., 1998). The phenotypes of *gpat4* and *gpat8* were indistinguishable from that of the WT throughout development, indicating putative redundancy between the *GPAT* genes (Figure 2D). Toluidine blue staining indicated high permeability of the leaf cuticle in *bdg1* and *dcr1-3*, showing severe defects in the barrier function of their cuticle layer (Figure 2D). However, the rosette of *gpat4* and *gpat8* did not show any toluidine blue staining of the shoot tissue, suggesting that the absence of a single *GPAT4* or *GPAT8* gene was not sufficient to disrupt the barrier integrity of the leaves, which is consistent with their lack of phenotype (Figure 2D). Interestingly, in the *gpat4* mutant, only the cotyledons turned blue, suggesting that the absence of GPAT4 might specifically affect the cuticle of the cotyledons (Figure 2D).

Next, we specifically investigated the effect of *bdg1* and *dcr1-3* mutants on the SAM cuticle using an Fluorol Yellow 088 fluorescent dye to stain lipophilic layers. We observed that the *bdg1* and *dcr1-3* mutants showed an uneven distribution of the cuticle, or even its absence in some places, compared to a thin and continuous staining for the WT (Figure 2E). In a second approach to remove the cuticle-like layer, we overexpressed *CUTICLE DESTRUCTING FACTOR 1* (*CDEF1*), which encodes a cutinase, specifically in the SAM (Takahashi et al., 2010). To achieve this, we generated a construct with *CDEF1* overexpression under the control of a *pML1* promoter that directs expression to the L1 layer of the SAM and epidermal cells (Supplemental Figure 5A) (Iida and Takada, 2021; Iida et al., 2023). The *pML1:CDEF1* transgenics displayed increased toluidine blue permeability and severe cuticle defects, including strong leaf fusion, similar to *bdg1* (Supplemental Figure 5A–5C). Indeed, the *pML1:CDEF1* plants showed slow growth, delayed or absent flowering, and periodic termination of growth suggesting that cutinase overexpression under the *ML1* promoter likely disrupted general plant growth and development (Supplemental Figure 5C). The phenotype of *pML1:CDEF1* plants exhibited leaf fusion similar to *bdg1* and *dcr1-3* but in a more pronounced manner (Supplemental Figure 5C).

To further explore cuticle integrity in the meristem of *bdg1* and *dcr1-3* cuticle mutants and *pML1:CDEF1* lines, we visualized toluidine blue permeability of shoot apices (Figure 2F and 2G). While WT shoot apices were not stained for toluidine blue, indicating an intact cuticle diffusion barrier, the meristems of the cuticle biosynthesis mutants stained blue, with more staining observed in *bdg1* than in *dcr1-3* (Figure 2G). The L1-specific expression of the CDEF1 cutinase led to highest accumulation of the toluidine blue dye in the meristem, suggesting the possible complete absence or severe destruction of the cuticle layer in this region by CDEF1 (Figure 2G). Next, we investigated how the disruption of the cuticular layer in the mutants and cutinase overexpression line affected maintenance of endogenous hypoxia in the SAM. Using oxygen microprofiling, we found that, in the WT, oxygen levels drop sharply in the SAM, but in *bdg1*, *dcr1-3*, and *pML1:CDEF1* plants this oxygen gradient was less steep. Indeed, oxygen levels were significantly higher in plants with a disrupted cuticle already at a depth of 10 μm within the SAM (Figure 2H).

To investigate how dynamic cuticle destruction of the SAM L1 layer affects oxygen levels we engineered a *pML1* tissue-specific dexamethasone (Dex)-inducible *CDEF1* line, taking advantage of a previously reported comprehensive two-component system for inducible, cell-type-specific expression in *Arabidopsis* (Schürholz et al., 2018). In this design, addition of the Dex inducer will translocate a *pML1*-driven *LhG4-GR* transcription factor to the nucleus, leading to activation of a synthetic *pOp6* promoter driving *CDEF1*, in addition to an mTurquoise2 reporter gene (Supplemental Figure 6A). After treating *Arabidopsis* seedlings with Dex, we successfully detected strong mTurquoise2 fluorescence in the L1 meristematic region and in young leaf primordia (Supplemental Figure 6B) and validated meristem permeability through toluidine blue staining, which clearly showed increased permeability in the SAM region, as well as in the leaf primordia (Supplemental Figure 6D). Successful Dex induction of this construct, as visualized by mTuquoise2, and enhanced SAM permeability to toluidine blue was also observed in the SAM of 4-week-old plants (Figure 2I and 2J; Supplemental Figure 6C). Together with leaf fusion phenotypes after 7–10 days, a typical cuticle-defect phenotype, showed that tissue-specific inducible expression of CDEF1 was effective in removing the SAM cuticle (Supplemental Figure 6E). Finally, oxygen microprofiling revealed a significant increase in oxygen content in the Dex-induced meristems compared to the ethanol-treated controls, suggesting that cuticle removal had a direct effect on oxygen levels in the SAM (Figure 2K). Taken together, the less steep oxygen profiles in cuticle-defective plants showed that an intact cuticle is necessary for the maintenance of chronic hypoxia in the SAM.

### Defective cuticle modifies hypoxia-responsive gene expression and meristem function

**Figure 3:**
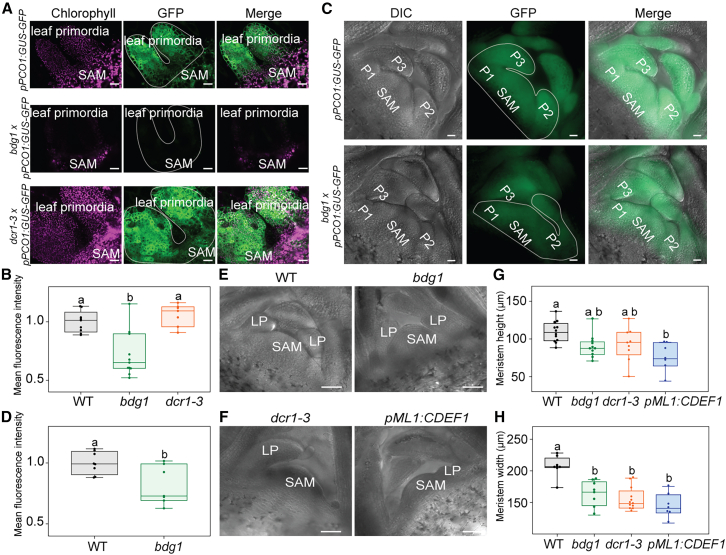
Hypoxia response in the SAM of cuticle biosynthesis mutants and its effect on SAM size. **(A)** Confocal images showing *PCO1* promoter activity driving GFP expression indicating hypoxia response in the SAMs and leaf primordia of WT, *bdg1*, and *dcr1-3 x pPCO1:GUS-GFP*, *pPCO1:GUS-GFP* 3-day-old seedlings. Magenta color represents the chlorophyll channel, green color represents GFP expression, and the merged image shows both. Scale bar, 20 μm (*n* = 8–11). **(B)** Mean fluorescence intensity values in the SAM and leaf primordia of *bdg1 x pPCO1:GUS-GFP*, *dcr1-3 x pPCO1:GUS-GFP*, and *pPCO1:GUS-GFP* 3-day-old seedlings (as indicated in the ROI in the images in **(A)** were measured using FIJI/ImageJ. Statistical differences were evaluated using one-way ANOVA followed by Dunnett’s test (*n* = 8–11). **(C)***pPCO1:GUS-GFP* confocal imaging in the vibratome sections of SAMs and leaf primordia of *bdg1 x pPCO1:GUS-GFP*, *pPCO1:GUS-GFP*, and WT 4-week-old plants. Scale bar, 20 μm (*n* = 7–8). **(D)** Mean fluorescence intensity values in the SAMs and leaf primordia of *bdg1 x pPCO1:GUS-GFP* and *pPCO1:GUS-GFP* 4-week-old plants (as indicated in the ROI in the images in **(C**) were measured using FIJI/ImageJ. Statistical differences were evaluated using a *t*-test (*n* = 7–8). **(E and F)** DIC images of the SAM vibratome sections of 4-week-old *bdg1*, *dcr1-3*, *pML1:CDEF1*, and WT plants. Section thickness, 120 μm. LP, leaf primordium. Scale bar, 50 μm (*n* = 7–12). **(G)** SAM height measurements of 4-week-old *bdg1*, *dcr1-3*, *pML1:CDEF1*, and WT plants were performed using Fiji/ImageJ. One-way ANOVA was performed, followed by Dunnett’s test for comparisons of each genotype to the WT (*n* = 7–12). **(H)** SAM width measurements of 4-week-old *bdg1*, *dcr1-3*, *pML1:CDEF1*, and WT plants were performed using Fiji/ImageJ. One-way ANOVA was performed, followed by Dunnett’s test for comparisons of each genotype to the WT (*n* = 7–12).

Previously it was shown that chronically low oxygen levels in the SAM lead to constitutive induction of hypoxia responses. To investigate whether the higher oxygen levels in cuticle mutants attenuate this, we crossed the *bdg1* and *dcr1-3* cuticle biosynthesis mutants with a *pPCO1:GUS-GFP* hypoxia-signaling reporter. Initial GUS staining results showed a stronger activation of this reporter in *bdg1* and *dcr1-3* seedlings, which was incoherent with higher internal oxygen levels (Supplemental Figure 7A). However, this appeared to be an artifact likely due to the increased permeability of cuticle mutants to the GUS staining solution, as pretreatments with a chloroform or acetone solvent, which removes the cuticle wax layer (Loneman et al., 2017), resulted in no significant differences in staining of the meristem area (Supplemental Figure 7A and 7B). These findings indicate that cuticle biosynthesis mutants are not suitable for qualitative or quantitative analyses using GUS-based reporters because of their altered cuticle permeability. Secondly, we visualized the activity of *pPCO1:GUS-GFP* in *bdg1* and *dcr1-3* by imaging the GFP signal in their SAMs. In *bdg1* seedlings, *pPCO1:GUS-GFP* signal intensity was significantly weaker compared to that in *dcr1-3* and WT plants, suggesting a reduced hypoxia response in the SAM (Figure 3A and 3B). To confirm whether lower *PCO1* promoter activity was due to higher oxygen levels in cuticle-defective plants, we visualized GFP signal intensity after a 3.03 kPa oxygen treatment. Under hypoxic conditions, the *pPCO1:GFP* signal in the SAM of *bdg1* was enhanced, indicating that the *PCO1* promoter remained responsive to oxygen levels (Supplemental Figure 7C and 7D). Similar results were observed with 4-week-old plants, although *bdg1* plants maintained moderate *pPCO1* activity at this stage (Figure 3C and 3D; Supplemental Figure 7E). Our findings indicate that the hypoxia response in plants with a disrupted cuticle is weakened but not abolished.

Next we investigated how the increase in oxygen content and attenuated hypoxia signaling in cuticle-defective plants affected plant development. First, we measured meristem width and height in *bdg1*, *dcr1-3*, and *pML1:CDEF1* lines in comparison with the WT (Figure 3E and 3F). Cuticle defects significantly affected meristem height in *bdg1* and *pML1:CDEF1* (Figure 3G) but not in *dcr1-3*. Meristem width was affected in all plants with an altered cuticle (Figure 3H). Thus, higher internal oxygen levels owing to a disrupted cuticle could have led to reduced meristem size. We next studied whether these changes relate to whole-plant growth. At the Netherlands Plant Eco-Phenotyping Center (NPEC), both *bdg1* and *dcr1-3* developed significantly smaller rosettes and showed increased rosette compactness compared with WT over a 35-day period (Supplemental Figure 8A–8D). To test whether cuticle removal and the associated increase in oxygen levels affected SAM development more directly, we examined the expression of genes associated with meristem function in *pML1:GR-LhG4:pOp4:CDEF1* after a Dex treatment of 2 and 4 days applied to shoot apices (Supplemental Figure 9A and 9B). A group of genes composed of several core meristem maintenance genes were mostly unaffected, with the exception of *BAM2*, whose downregulation is associated with reduced meristem size and delayed growth (Supplemental Figure 9C and 9D) (DeYoung et al., 2006). A second group of genes associated with meristem patterning and boundary specification showed a more consistent downregulation. *REVOLUTA* (*REV*), a class III homeodomain-leucine zipper (HD-ZIP) transcription factor, was downregulated on the fourth day of cuticle degradation, together with *LITTLE ZIPPER1* (*ZPR1*) and *ARABIDOPSIS THALIANA HOMEOBOX GENE 1* (*ATH1*) (Supplemental Figure 9E and 9F). Together, these results suggest that cuticle removal affects *BAM2* in addition to genes involved in organ patterning and boundary identity, potentially contributing to the distinct phenotype observed in the cuticle biosynthesis mutants and cutinase overexpression line. It remains to be determined whether this outcome is driven by elevated oxygen levels or primarily by cuticle absence.

### Mitochondrial respiration and resource availability affect internal SAM oxygen levels

**Figure 4:**
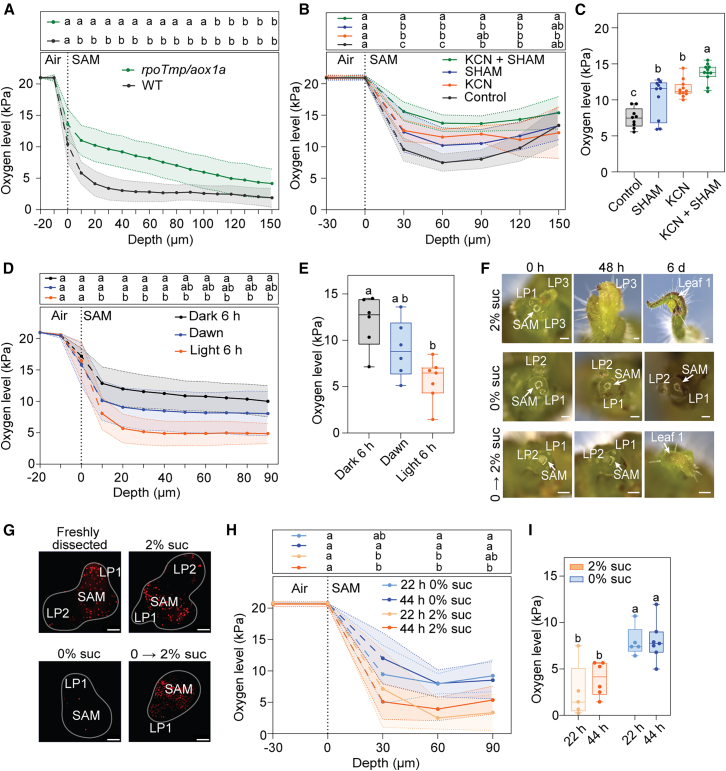
The effect of mitochondrial disfunction and resource availability on oxygen levels in the SAM. **(A)** Oxygen measurements in the SAMs of double *rpoTmp* (*SALK_132842*)*/aox1a* (*SALK_084897*) *Arabidopsis* plants measured with a Clark-type oxygen microsensor with a 3- to 5-μm tip. Oxygen levels were measured every 10 μm to a depth of 150 μm. The vertical stippled line denotes the estimated position of the SAM boundary using microscopy. The dashed segment of the oxygen microprofile indicates the transition of the sensor across this boundary, where oxygen levels are less certain. Statistical differences were evaluated using two-way repeated-measures ANOVA, followed by Šídák’s multiple-comparisons test (at each depth), *p* < 0.05 (*n* = 8). **(B)** Oxygen microprofiling in the dissected SAMs of 10-day-old tomato seedling performed using a Clark-type oxygen microsensor after 30-min incubation in 2 mM KCN, 10 mM SHAM, or the combined media. Oxygen levels were measured every 30 μm up to a depth of 150 μm. The vertical stippled line denotes the estimated position of the SAM boundary using microscopy. The dashed segment of the oxygen microprofile indicates the transition of the sensor across this boundary, where oxygen levels are less certain. Statistical differences were evaluated using two-way repeated-measures ANOVA, followed by Tukey’s multiple-comparisons test (at each depth), *p* < 0.05 (*n* = 9–12). **(C)** Clark-type oxygen measurements comparing dissected tomato meristems incubated for 30 min in 2 mM KCN, 10 mM SHAM, or the combined media at 60-μm depth. Statistical differences were evaluated using two-way ANOVA followed by Tukey’s test, *p* < 0.05 (*n* = 9–12). **(D)** Clark-type oxygen measurement profiles showing oxygen profiles in *Arabidopsis* SAM at dawn, 6 h into the light period, and after an extended night of 6 h. The vertical stippled line denotes the estimated position of the SAM boundary using microscopy. The dashed segment of the oxygen microprofile indicates the transition of the sensor across this boundary, where oxygen levels are less certain. Statistical differences were evaluated using two-way repeated-measures ANOVA, followed by Tukey’s multiple-comparisons test (at each depth), *p* < 0.05 (*n* = 6–7). **(E)** Clark-type oxygen measurements at a depth of 30 μm in the *Arabidopsis* SAM at dawn, after 4 h in light, and after an extended night of 6 h. Statistical differences were evaluated using two-way ANOVA followed by Tukey’s test, *p* < 0.05 (*n* = 6–7). **(F)** Microscopic images of tomato SAMs after dissection, 48 h, and 6 d of growth on 2% sucrose medium, on medium without sucrose, and transferred from medium without sucrose to medium with 2% sucrose after 48 h. Scale bar, 200 μm. **(G)** Confocal maximum intensity projection images showing active cell division following EdU incorporation and Alexa Fluor 594 labeling. Each red dot represents S-phase cell-cycle progression. The SAM is indicated with a drawn line. Scale bar, 50 μm. **(H)** Clark-type oxygen measurement profiles comparing dissected meristems grown on media with and without sucrose after 22 and 44 h. The vertical stippled line denotes the estimated position of the SAM boundary using microscopy. The dashed segment of the oxygen microprofile indicates the transition of the sensor across this boundary, where oxygen levels are less certain. Statistical differences were evaluated using two-way repeated-measures ANOVA, followed by Tukey’s multiple-comparisons test (at each depth), *p* < 0.05 (*n* = 5–7). **(I)** Clark-type oxygen measurements comparing dissected meristems grown on media with and without sucrose at 60-μm depth after 22 and 44 h. Statistical differences were evaluated using two-way ANOVA followed by Tukey’s test, *p* < 0.05 (*n* = 5–7).

It has been hypothesized that high oxygen consumption rate via respiration is a key factor in driving endogenous hypoxia in plant tissues (van Veen et al., 2025). To confirm that mitochondrial respiration contributes to oxygen consumption in the SAM, we used the *Arabidopsis rpoTmp/aox1a* double mutant (Kühn et al., 2015). The *rpoTmp* surrogate mutation removes one of the two bacteriophage-type RNA polymerases in mitochondria and causes a strong reduction in mitochondrial gene transcription, leading to an 85% decrease in the activity of complexes I and IV (Kühn et al., 2009). The *aox1a* mutation eliminates the ALTERNATIVE OXIDASE 1A (AOX1A) isoform, which helps maintain electron flow during stress or when cytochrome *c* oxidase is inhibited as is the case in *rpoTmp* (Giraud et al., 2008; Merendino et al., 2020). Therefore, by combining both mutations, we aimed to reduce oxygen-consuming processes as much as possible. The *rpoTmp/aox1a* SAM showed a significantly higher oxygen level in the meristem core compared to the WT, but the values became comparable to the WT in the very deep layers of the SAM (Figure 4A). As a complementary approach, we directly targeted mitochondrial respiration by inhibiting both oxidative phosphorylation and the alternative oxidase pathway in the SAMs of tomato seedlings. Potassium cyanide (KCN) was used to block complex IV of the electron transport chain, while salicylhydroxamic acid (SHAM) specifically inhibited the activity of the alternative oxidase (AOX). Oxygen levels in the SAM increased significantly and to a similar extent when either complex IV was inhibited with KCN or AOX activity was blocked with SHAM (Figure 4B and 4C). When applied simultaneously, the inhibitors had an additive effect that resulted in an even higher oxygen level in the SAM (Figure 4B and 4C), suggesting that AOX1a and the cytochrome *c* oxidase pathway may functionally compensate for each other under inhibitory conditions in the SAM. These results from the rpoTmp/aox1a mutant and chemical inhibitor treatments indicate that mitochondrial respiration contributes to the establishment of hypoxia in the SAM.

Next, we asked whether respiratory oxygen consumption is linked to active cell proliferation. Plants depend on glucose to sustain growth and metabolic processes, but resource availability can become limited in sink tissue, particularly at night (Triozzi et al., 2024). To assess how sugar depletion affects oxygen consumption, we measured oxygen levels in *Arabidopsis* SAMs at dawn, when starch reserves were depleted by respiration in the night (Graf et al., 2010), and compared them to SAMs from plants either re-exposed to 6 h of light after dawn or subjected to a +6 h extended night to induce starvation (Figure 4D and 4E). Our results revealed that the SAM has higher oxygen levels at dawn, or when subjected to starvation, likely owing to suppressed metabolic activity due to sugar limitation or a lack of light (Figure 4D and 4E). In addition to resource availability, it is known that SAM activity depends on light as a growth stimulus, through activation of cytokinin biosynthesis (Yoshida et al., 2011). Consequently, even with sufficient glucose, SAM activity may be suppressed in the dark. We, therefore, sought to validate whether the increased oxygen levels in the SAM were primarily due to sugar limitation rather than the absence of light stimuli. Tomato SAMs were chosen for this experiment because they can be easily dissected and grown in culture (Hamant et al., 2019). We dissected tomato SAMs, removed all surrounding leaves, leaving only few emerging primordia, and cultured them on media with or without sucrose but in the presence of light (Figure 4F). As expected, meristems grown on media without sucrose showed no new leaf primordia initiation, whereas those grown in sucrose-containing media produced new leaves (Figure 4F). To confirm that the meristems grown without sucrose remained viable, they were transferred to sucrose-containing media after 48 h, which reactivated organogenesis (Figure 4F), indicating that the meristems had likely entered a state of quiescence, but did not terminate on the non-sucrose-containing medium. To validate meristem quiescence, that is, a lack of cell division, we conducted an 5-ethynyl-2′-deoxyuridine (EdU) DNA proliferation assay. Similar to fresh-dissected meristems, SAMs grown for 24 h on sucrose-containing media showed active cell division, whereas those grown without sucrose had very few dividing cells (Figure 4G). Additionally, when the meristems grown without sucrose for 24 h were transferred to sucrose-containing media, they exhibited clear reactivation of cell division (Figure 4G).

After confirming that we could modify SAM activity through exogenous sucrose supplementation, we then measured its effect on internal oxygen levels. After 22 h of growth without sucrose, oxygen levels in the SAM increased, reaching up to 7 kPa, compared to 4 kPa on sucrose (Figure 4H and 4I). To determine whether oxygen concentrations would continue rising or instead stabilize due to residual metabolic activity, tissue compactness and the cuticle barrier, we also measured SAM oxygen levels after 44-h sucrose deprivation (Figure 4H and 4I). No significant difference was observed between 22 h and 24 h without sucrose , suggesting that the SAM maintains a relatively stable, albeit somewhat higher, internal oxygen concentration even under extended carbon starvation. To verify the effect of sugar availability on meristem metabolism, we measured respiration rate of the meristems grown with and without sucrose using a Unisense nanorespiration setup (Supplemental Figure 10A). As expected, the respiration rate of the actively dividing SAM in the presence of sucrose was higher than that of the quiescent SAM (Supplemental Figure 10B). However, despite the absence of sucrose in the medium, quiescent SAMs continued to consume a substantial amount of oxygen (Supplemental Figure 10B). Hence, we reasoned that photosynthesis could contribute to internal oxygen levels in the SAM, either via the direct release of oxygen or the production of sugars to fuel respiration. To investigate the effect of photosynthesis by the meristem or adjacent tissues, we aimed to reduce chlorophyll pigments. We, therefore, cultivated dissected tomato meristems on medium supplemented with sucrose and norflurazon, a herbicide that inhibits chlorophyll biosynthesis (Supplemental Figure 10C). After 3 days, the meristems turned almost completely white, indicating effective chlorophyll depletion (Supplemental Figure 10C and 10D). However, when we performed a norflurazon treatment on medium without sucrose, the meristems failed to turn white. To overcome this, we first grew the dissected meristems for 48 h on medium containing both sucrose and norflurazon and then transferred them to medium without sucrose to arrest cell proliferation (Supplemental Figure 10C). Despite the absence of pigments, we did not observe changes in oxygen concentration in norflurazon-treated meristems, indicating that even chlorophyll-depleted, inactive shoot apices still maintain relatively low oxygen levels (Supplemental Figure 10E and 10F). The persistent low oxygen levels (7–10 kPa) in quiescent meristems imply that maintenance respiration, together with tissue compactness and the presence of the cuticle barrier, act to stabilize chronic hypoxia by restricting oxygen diffusion into the SAM.

### Reaction-diffusion model of oxygen in the SAM reveals different roles for compactness, the cuticle, and respiration rate

**Figure 5:**
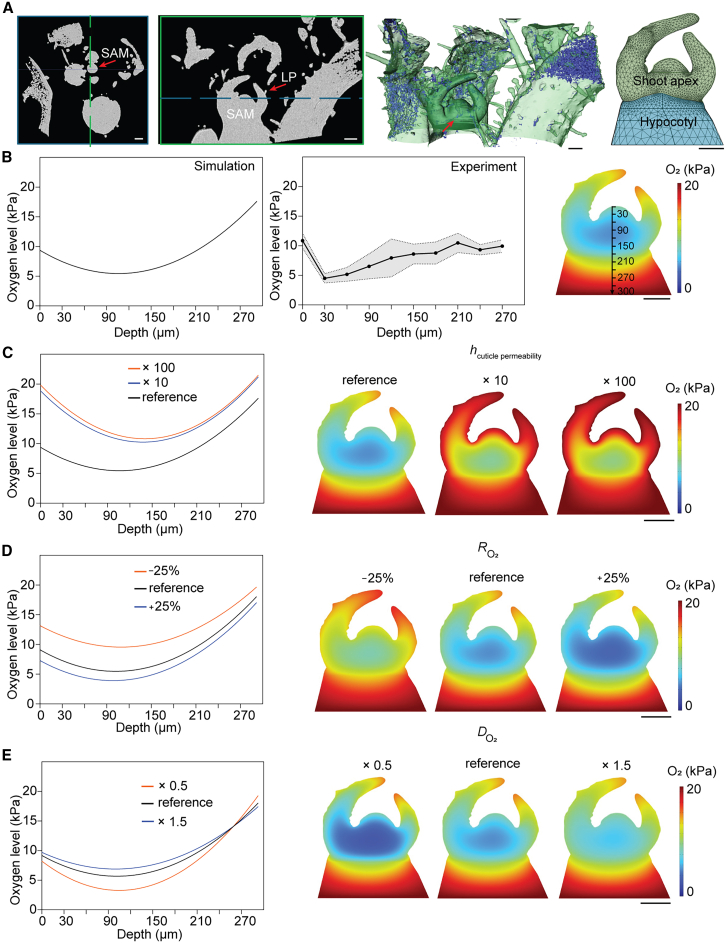
A reaction-diffusion model of oxygen distribution in the apical meristem. **(A)** Geometrical model development and porosity analysis (from left to right): μCT imaging analysis, showing two orthogonal slice views of the meristem, indicated with the arrow (scale bar, 100 μm); 3D surface rendering (in green) of the μCT scan. Any internal air spaces are shown in blue: the SAM and subapical zone with leaf primordia have zero porosity; 3D geometric model of the meristem ROI, used for oxygen transport modeling. **(B)** Model simulation of oxygen transport in the meristem compared to measurements (from left to right): simulated oxygen partial pressure distribution in meristem for the reference values of model parameters h_cuticle_ = 1 × 10^−7^ m s^−1^, R_O2_ = 1 × 10^−2^ mol m^−3^s^−1^, and D_O2_ = 5 × 10^−11^ m^2^ s^−1^; oxygen partial pressure profile through the meristem in the apical-to-basal direction along the arrowed axial line obtained with a Clark-type oxygen microsensor, comparing model simulations (middle) and experiments (right, *n* = 4, spread is standard deviation). **(C–E)** Sensitivity of oxygen profiles to model parameters (showing distribution contours and axial line profiles). **(C)** Sensitivity to cuticle permeability. **(D)** Sensitivity to maximum respiration rate. **(E)** Sensitivity to diffusivity due to changes in porosity.

We so far investigated the effects of three individual factors on endogenous oxygen levels in the SAM. Each of these factors (tissue compactness, cuticle, and respiration rate) contributes to the hypoxic microenvironment. However, evaluating their combined impact and quantifying the contribution of each factor remained challenging. To tackle this, we used a steady-state reaction-diffusion model to simulate oxygen transport across the SAM and surrounding tissues, and compared the output of this model with experimentally measured values (Supplemental Table 1). The simulation geometry model, including porosity values for the SAM, leaf primordia, and hypocotyl, were extracted from μCT scans of tomato seedlings (Figure 5A). The diffusivity of oxygen within the SAM was assumed to equal that in water because of the absence of intercellular airspaces in the μCT data (Figure 1G and 1H). Respiration rates were experimentally determined using dissected tomato meristems approximately 250 μm in size, corresponding to the meristem itself (∼200 μm) plus a small portion of the hypocotyl (Supplemental Figure 11A; Supplemental Table 1). As the respiration rates were determined using single shoot apices, these data were converted into volumetric respiration rate values by calculating the average volume of the SAM based on 3D confocal images using membrane staining (FM4-64) (Supplemental Figure 11B and 11C). Given the high cell density and metabolic activity in SAMs, we assumed that their respiration rates per unit volume exceed those of more vacuolated tissues such as the hypocotyl (Supplemental Table 1). Boundary conditions included oxygen diffusion through the cuticle and diffusion into the hypocotyl (Supplemental Table 1). The cuticle was set to cover both the meristem and leaf primordia in a similar manner, supported by Fluorol Yellow staining in *Arabidopsis* (Figure 2A). We also assumed that oxygen production within the tissue was negligible, based on previous experiments showing no change in oxygen levels upon chlorophyll depletion (Supplemental Figure 10E and 10F). The simulation reproduced a steep oxygen gradient from the meristem surface toward its core, with oxygen levels increasing again in the hypocotyl (Figure 5B). These results matched the oxygen profiles measured with a Clark-type oxygen microsensor (Figure 5B). Moreover, the model predicted low oxygen concentrations in leaf primordia, consistent with experimental data (Figure 5B; Supplemental Figure 11D and 11E).

We next explored the sensitivity of the model to modifications of the three factors we experimentally found to be important for internal SAM hypoxia. A 10-fold increase in cuticle permeability strongly reduced the gradient steepness but maintained a moderate hypoxic core 120 μm deep within the meristem (Figure 5C). Experimental data showed that oxygen levels drop rapidly in the first layers of the meristem, unless the cuticle was genetically disrupted, and hypoxia-signaling reporters were activated throughout the SAM (Figures 2 and 3). Hence, the presence of a strong cuticle gas barrier is supported both experimentally and by modeling. Both meristem models with a 10- and 100-fold more permeable cuticle could still maintain a hypoxic core of ∼10- to 12-kPa oxygen, suggesting that tissue porosity and respiration still play major roles in maintaining hypoxia (Figure 5C).

To assess the importance of respiration, we tested the effect on the model when we modified its rate. A 25% increase in respiration lowered oxygen levels throughout the SAM, while a 25% reduction in meristem respiration substantially elevated internal oxygen levels, showing that respiration is an important factor for determining the extent of hypoxia (Figure 5D). Experimentally, a similar reduction in respiration induced by mitochondrial disfunction and sucrose limitation led to a comparable rise in SAM oxygen levels (Figure 4A, 4C, and 4I). Moreover, simulations with drastically reduced respiration (10-fold less) required a ∼100-fold lower cuticle permeability (compared to literature values for aquatic plant cuticles) to achieve hypoxic conditions (Supplemental Figure 12A).

Simulations in which we tested the sensitivity of the model to different diffusivity values showed that using values scaled to ×0.5 or ×1.5 relative to the reference of 5 × 10^−11^ m^2^ s^−1^ altered SAM core hypoxia but had only a minor impact on oxygen levels in outermost cell layers (Figure 5E). Further increased diffusivity homogenized oxygen levels withing the meristem and leaf primordia (Supplemental Figure 12B). Even under extreme diffusivity values (1000-fold) that are comparable to leaf tissue, the SAM did not reach atmospheric oxygen levels, indicating that respiration and the cuticle barrier can sustain low oxygen levels even if the SAM is highly porous (Supplemental Figure 12B).

Finally, we simulated the effect of tissue-specific differences in respiration rate within the SAM. We therefore modified the reaction-diffusion model to create subdomains for the central zone (CZ), organizing center (OC), peripheral zone (PZ), and primordia (Supplemental Figure 13A). To test how heterogenous metabolism in the SAM would affect internal oxygen distribution, we assumed in our updated model the respiration rate of the tissue-region subdivisions to be proportional to the division rates in the tissue region (Reddy et al., 2004; Kitagawa and Jackson, 2019) (Supplemental Tables 2 and 3). Comparing the updated model with the original model, we found that the oxygen concentration in the updated model was approximately 2 kPa higher in the CZ and OC, yet the distribution was largely unchanged compared to the original model and still comparable to the experimental oxygen profiles (Supplemental Figure 13). We acknowledge that the quantitative connection between cell division rate and tissue-specific respiration in SAMs is hypothetical. Future research should seek to quantify the tissue-specific respiration kinetics to more conclusively identify the role of the tissue zones in establishing the hypoxic niche in the SAM.

In conclusion, the meristem modeling data demonstrated that the extent of hypoxia is primarily driven by high respiration rates and maintained by limited oxygen diffusion, resulting from compact tissue structure and the presence of a cuticle.

## Discussion

Hypoxia is a conserved feature of stem cell niches across kingdoms and is observed in both animals and plants. In animals, stem cells are often embedded in low-oxygen microenvironments, where hypoxia supports pluripotency and regulates differentiation (Huang et al., 2018). In plants, the SAM governs stem cell maintenance and organogenesis and has also been identified as a hypoxic niche (Weits et al., 2019). Our findings suggest that this hypoxic condition is not coincidental but more likely an actively maintained state through multiple contributing factors. This stable low-oxygen environment supports the activity of oxygen-labile proteins such as LITTLE ZIPPER 2 (ZPR2) and VERNALIZATION 2 (VRN2), which are important for leaf initiation and the transition to flowering (Weits et al., 2019; Osborne et al., 2025). While pleiotropic effects of a disrupted cuticle cannot be ruled out, our study provides interesting observations to further investigate the causal relationship between cuticle integrity, internal oxygen levels, and downstream developmental processes. For instance, increased oxygenation would lead to proteolysis of ZPR2 and VRN2. Although ZPR2 acts at the post-translational level, one of its primary targets, the HD-ZIP III transcription factor REV was also found to be transcriptionally downregulated upon cuticle destruction (Supplemental Figure 9F). This downregulation occurred together with ZPR1, which, as ZPR2, functions in a negative-feedback loop with REV (Wenkel et al., 2007; Kim et al., 2008) (Supplemental Figure 9F). HD-ZIP III proteins are key regulators of apical-basal polarity, organ initiation, and vascular patterning, and REV in particular contributes to proper meristem patterning, axillary meristem formation, and organ positioning (Emery et al., 2003; Byrne, 2006; Caggiano et al., 2017). In addition, *ATH1*), a homeobox gene involved in organ separation and stem formation, also showed reduced expression after 4 days of cuticle degradation (Supplemental Figure 9F). Reduced *REV*, *ZPR1*, and *ATH1* expression suggests that information about organ position within the SAM may be compromised when internal oxygen levels rise (Supplemental Figure 9E and 9F). We recognize that targeted destruction of the cuticle will likely affect mechanical stability of the SAM, and mechanical signals have, in turn, been shown to regulate developmental patterning (Onoda et al., 2012; Landrein and Ingram, 2019). Future studies should aim to disentangle these mechanical effects on plant development from the impact of meristem oxygenation due to cuticle disruption.

This work and previous studies place low oxygen alongside other stress-associated signals that influence developmental processes and are tightly regulated within the SAM. For example, reactive oxygen species, which are usually linked to stress, including acute hypoxia, help define the boundary between the stem cell region and the differentiation zone. If this redox balance is disturbed, meristem activity is lost (Zeng et al., 2017). In addition, ethylene, a gaseous hormone known for its roles in fruit ripening, senescence, abscission, and environmental stress responses, also plays a key developmental role in the SAM. Mutants that cannot respond to ethylene fail to maintain stem cell identity and show early meristem abortion (Zeng et al., 2021). How oxygen distribution, gaseous barriers, and respiration interplay with reactive oxygen species and ethylene levels in the SAM is an exciting topic for future studies.

In order to study how low oxygen is involved in SAM development, it is crucial to understand how the hypoxic state of the SAM is sustained and what factors influence its internal oxygen levels. Here, we show that a combination of restricted oxygen entry, limited diffusivity, and high metabolic demand establishes and sustains hypoxia in the SAM. Using X-ray μCT and reporter lines, we found that tissues surrounding the SAM are more porous and exhibit higher oxygen levels, suggesting that the compactness of SAM tissue reduces gas diffusivity and helps maintain hypoxia (Figure 1). This is supported by the sensitivity of the modeled gas distribution to diffusivity (Figure 5). We also demonstrated that the cuticle serves as a physical barrier limiting oxygen diffusion into the SAM. Genetic disruption of proper cuticle biosynthesis or direct enzymatic cuticle degradation resulted in increased oxygen levels and a decreased hypoxia response (Figures 2 and 3). While the cuticle is traditionally understood as a barrier against water and oxygen loss (Fich et al., 2016), our findings highlight a new role of keeping oxygen out and therefore sustaining hypoxia in the SAM (Figure 2).

High respiration is often a key driver of a strong hypoxia response, as shown in tissues after *Botrytis cinerea* infection or crown gall formation (Kerpen et al., 2019; Valeri et al., 2021). Oxygen measurements at the end of night, when starch reserves are low, showed higher internal oxygen levels than after 6 h of light, revealing potential circadian regulation of oxygen levels in the SAM, next to previously found cyclic hypoxia in leaves (Triozzi et al., 2024). Next, we linked oxygen dynamics in the SAM to its metabolic state. SAMs that actively produce leaf primordia under sucrose supply show lower oxygen levels than quiescent SAMs, suggesting that metabolic activity contributes to the formation of hypoxia (Figure 4). However, while the SAM gas distribution model showed that strongly (10-fold) reduced respiration rates lead to almost aerobic levels in the SAM, our experimental measurements showed that oxygen remained relatively low (∼7 kPa) even when primordia were no longer initiated at the SAM (Figure 4; Supplemental Figure 12A). In the model sensitivity tests, only a 100 times more impermeable cuticle could maintain SAM hypoxia when respiration rates were significantly decreased (Supplemental Figure 12A). Thus, either the SAM cuticle is a much more effective barrier than we assumed in the model or residual metabolic activity continues to consume oxygen, sustaining hypoxia even in growth-arrested conditions. The latter is supported by our nanorespiration measurements, as meristems grown without sucrose still consumed oxygen (Supplemental Figure 10B). Hence, residual oxygen consumption by non-proliferative maintenance processes could prevent full equilibration with ambient air (Amthor, 2000). We may speculate that oxygen consumption observed during the inactive state of the SAM might result from the breakdown of sugars, amino acids, or cellular components of the meristem itself or its surrounding tissues. This could support basic metabolic functions such as nutrient transport, protein turnover, and maintenance of ion gradients (Amthor, 2000). Maintenance respiration, together with low diffusivity and the cuticle barrier, may thus contribute to stable low oxygen levels in the SAM. Such homeostatic oxygen levels might be required to prevent ZPR2 and VRN2 proteolysis. Moreover, hypoxia may be a required condition for proliferation, similar to animals where low oxygen is required to preserve pluripotency and prevent uncontrolled differentiation (Ezashi et al., 2005).

We were not able to experimentally test the combined effect of all three factors contributing to SAM hypoxia or determine specific contributions of each of them. To tackle this, we used a reaction-diffusion model, which not only confirmed the sensitivity of oxygen levels to changes in each factor but also helped define their specific contributions (Figure 5). We concluded that tissue compactness is most critical for maintaining a controlled oxygen level in the deep layers of the SAM. The cuticle is required to establish a steep oxygen gradient between the surface and the outermost layer of the SAM. Respiration initiates the overall reduction of internal oxygen levels and determines the extent of hypoxia in the whole meristem.

Interestingly, hypoxic niches are remarkably resilient to low oxygen, more than tissues that are not chronically hypoxic (Rankenberg et al., 2024). This resilience could come from a shifted metabolism that already prepares the tissue to cope with acute hypoxic stress, or the SAM is adapted to maintain ATP production via respiration at low oxygen availability, when other tissue induce a shift toward fermentative metabolism (Zabalza et al., 2009). Understanding how meristems establish and cope with chronic hypoxia could lead to applications in other research such as improving flooding tolerance, controlling developmental transitions, growth rate, and enhancing overall plant performance. For instance, dissecting the metabolism of the chronically hypoxic SAM may reveal metabolic or antioxidant pathways that can be engineered to confer resilience under acute hypoxia (flooding). Moreover, identifying the three key features underlying chronic hypoxia in the SAM provides a framework to predict when and where oxygen gradients are likely to arise elsewhere in the plant, enabling targeted investigation of their physiological and developmental roles.

Together, our findings offer an explanation of how developmental niches can sustain hypoxia through a combination of a diffusion barrier, tissue density, and metabolic activity. These factors may extend to other hypoxic tissues found in plants. A comparable niche is the initiating root primordium, which shows a strong hypoxic response and is also covered by a cuticle at the tip (Berhin et al., 2019; Shukla et al., 2019). This tissue also undergoes rapid proliferation, which may trigger hypoxia, leading to the stabilization of ERF-VII, which promotes the transition to a mature lateral root program. The causes and consequences of other low-oxygen niches such as maize kernels, anthers, fruit, callus regeneration, and grape buds are also under investigation (Kelliher and Walbot, 2014; Meitha et al., 2015; Langer et al., 2023; Koo et al., 2024; Xiao et al., 2024). Understanding how such chronic hypoxic niches are maintained and potentially regulated by endogenous hypoxia may provide new tools for improving traits such as flooding tolerance, developmental control, and growth rate in crops.

## Methods

### Plant materials

Seeds of two plant species were used: *A. thaliana* Columbia-0 (Col-0) and *S. lycopersicum* dwarf cultivar Micro-Tom. Col-0 was used as WT ecotype in the experiments with mutants *gpat4* (N667613), *gpat8* (N595122), *bdg1* (N861713), *dcr1-3* (N865435), and *clv3-15* (N68824), *rpoTmp* (SALK_132842)/*aox1a* (SALK_084897). *Gpat4*, *gpat8*, *bdg1*, and *dcr1-3* seeds were obtained from the Nottingham Arabidopsis Stock Center and genotyped (Supplemental Figure 4). The primers used in genotyping are listed in Supplemental Table 4. *pBDG1:GFP*, *pDCR:GFP*, *pGPAT4:GFP*, and *pGPAT8:GFP* were previously described in Berhin et al. (2019) and provided by the authors (Berhin et al., 2019). *clv3-15* seeds were kindly provided by Assistant Professor Dr. Marcel Proveniers (Forner et al., 2015). *rpoTmp*/*aox1a* seeds were kindly provided by Dr. Livia Merendino (Kühn et al., 2015; Merendino et al., 2020). Pollen of *pPCO1:GFP-GUS* homozygous plants was used to fertilize cuticle biosynthesis mutants *bdg1*, *dcr1-3*. The genotype *pPCO1:GFP-GUS* is described in Weits et al. (2014). The genotypes *pHRPEx5:GFP-GUS* and *pHRPEx5:UnaG-mCherry* are described in Panicucci et al. (2020). The seeds of the *pML1* driver line were provided by the authors (Schürholz et al., 2018).

### Construct design and production

The *pML1:CDEF1* construct was designed using GreenGate cloning (Lampropoulos et al., 2013). First, the *ML1* promoter was amplified from Col-0 genomic DNA and cloned into the T0 GreenGate pGGA000 entry module using BsaI restriction sites. Second, the *CDEF1* coding sequence was cloned into the T0 GreenGate pGGC000 entry module using BsaI recognition sites. The GreenGate reaction, containing 100 ng of pML1 PGGA000 (promoter), pGGB003 (dummy N-tag), CDEF1 (CDS), pGGD003 (mCherry C-terminal fusion tag), pGGE009 (UBQ10 terminator), pGGF005 (hygromycin resistance cassette), and pGGZ003 (destination vector) entry modules, 1 μl of BsaI fast digest (Thermo Fisher Scientific), and 2.5 μl of Anza T4 DNA Ligase Master Mix (Thermo Fisher Scientific) in a final volume of 10 μl, was performed in a thermocycler with 30 cycles of 37°C for 2 min and 16°C for 2 min, followed by 50°C for 5 min and 80°C for 5 min. Destination vectors were tested by restriction and sequencing.

To create Dex-inducible *CDEF1* expression in the pML1 region, the driver-effector toolkit was implemented based on the study from Schürholz et al. (2018). The existing pML1 driver line was transformed with the generated *pOp4:CDEF1* plasmid constructed using GreenGate cloning. The pML1 driver line was made using GreenGate cloning with two expression cassettes on one T-DNA. One cassette contains the promoter of *ML1* driving chimeric GR-LhG4 transcription factor and the second cassette contains *pOp6* promoter driving mTurquoise2 fluorescent reporter. The effector line is designed using the *pOp6* promoter that was cloned into the T0 GreenGate pGGA000 entry module using BsaI restriction sites. Due to a bacterial recombination issue, the final verified sequence consisted of four *pOp* repeats instead of six. The final GreenGate reaction contained 100 ng of pOp4 PGGA000 (promoter), pGGB003 (dummy N-tag), CDEF1 PGGC000 (CDS), pGGD003 (dummy C-tag), pGGE009 (UBQ10 terminator), pGGF005 (hygromycin resistance cassette), and pGGZ003 (destination vector) entry modules. Destination vectors were tested by restriction and sequencing. The primers used in cloning are listed in Supplemental Table 5.

### Transgenic plant generation

The constructs were introduced into Col-0 using the flowering dipping method (Clough and Bent, 1998). Transgenic plants were screened on agarized medium with the appropriate antibiotic.

### Growth conditions

*A. thaliana* seeds for *in vitro* cultivation were sown on half-strength, agarized Murashige and Skoog medium (pH 5.7) (Murashige and Skoog, 1962) after 3 days of vernalization at 4°C in the dark. Seeds were then germinated in short-day conditions (9 h light/15 h dark, 20°C, 70% relative humidity, 140 μmol photons m^−2^ s^−1^). Plants were grown vertically. Four-day-old seedlings were used for Flourol Yellow staining and GFP imaging, and 7-day-old seedlings were used for GUS staining imaging.

For experiments that required Micro-Tom plants or older Col-0 plants, such as for genotyping, phenotyping, oxygen measurements, and staining, plants were grown on Primasta soil mix. Micro-Tom seeds were placed in the climate chamber directly after sowing, and Col-0 seeds were first stratified in the dark for 3 days before being transferred to a short-day conditions (9 h light 15 h dark, 20°C, 70% relative humidity, 140 μmol m^−2^ s^−1^ light intensity).

Plants growth at the NPEC with the fully automated plant phenotyping module Helios were maintained under light, temperature, and humidity settings matching those described above.

### Confirmation of T-DNA insertion knockout lines

For screening of homozygous single insertion transgenic lines, the genomic DNA was extracted from the leaf tissue of WT, *bdg1*, *dcr1-3*, *gpat4*, and *gpat8* by grinding tissue in DNA extraction buffer (200 mM Tris–HCl pH 7.5, 250 mM NaCl, 25 mM EDTA, and 0.5% SDS), precipitating DNA with isopropanol, and resuspending DNA in water. All genomic DNA samples were PCR screened using left and right genomic primers (LP and RP, respectively) and the T-DNA border primer (LB) specific for each T-DNA insertion. The primers used in genotyping are listed in Supplemental Table 4.

### GUS staining

GUS staining of plants expressing the *PCO1* and *HRPEx5* promoters fused to *GUS-GFP* was performed by 2-hour incubation at 37°C with GUS-staining solution (100 mM buffer phosphate, 0.1% Triton X-100, EDTA pH 8 10 mM, potassium ferrocyanide 0.5 mM, potassium ferricyanide 0.5 mM, X-Gluc 200 mM) and then cleared in 70% (v/v) ethanol. Images were obtained using the Leica THUNDER imager model organism. FIJI/ImageJ was used to quantify the GUS expression intensity.

### Fluorol Yellow staining

Vertically grown (in ½ MS-agar plates) 4-day-old *Arabidopsis* or Micro-Tom seedlings were incubated in Fluorol Yellow 088 (0.001% w/v, in methanol) (Sigma) for 10 min and rinsed in water (two baths of 5 min each). The plants were mounted on slides for microscope examination. The cuticle-like layer was detected using confocal laser scanning microscopy Zeiss Airyscan 800 using a GFP channel (excitation 488 nm, detection 410–575 nm).

### FM4-64 membrane staining

For visualization of the meristematic tissue in 3- to 4-day-old Col-0 seedlings, cotyledons were removed using tweezers, and the seedlings were immersed in FM4-64 solution (50 μg/ml, prepared by diluting 5 μl of 1-mg/ml stock in 100 μl of distilled water). Incubation was performed in the dark for 10 min, followed by a single rinse with distilled water. Imaging was performed using a Zeiss Airyscan 800 confocal microscope with excitation at 506 nm and detection between 547 and 640 nm.

For volume measurements of vegetative Micro-Tom meristems, samples were first stained in an Eppendorf tube with FM4-64 solution (50 μg/ml, prepared by diluting 5 μl of a 1-mg/ml stock in 100 μl of distilled water) for 10 min followed by a single rinse with distilled water. Meristems were imaged on agarized plates using a 40× water-dipping objective on a Zeiss LSM 900 confocal microscope, with excitation at 506 nm and detection between 547 and 640 nm.

### Toluidine blue staining

In order to visualize the cuticle defects, a toluidine blue (TB) test was performed (Tanaka et al., 2004). For TB visualization in a whole-plant rosette, 3-week-old plants were submerged in aqueous solution of 0.05% (w/v) TB at room temperature. After 5–10 min, the TB solution was removed and plants were washed gently with water to remove excess TB from the plants.

Dex-inducible expression of CDEF1 was tested using 0.05% TB staining. Ten-day-old seedlings were incubated in the staining solution and rinsed with water prior to vibratome sectioning.

For TB visualization in the SAM of 4-week-old plants, an aqueous solution of 0.2% (w/v) TB was applied on the apices of the rosettes. After 20 min, the TB solution was removed and plants were washed gently with water prior to vibratome sectioning. The staining was visualized using Leica M205 FCA widefield microscope.

### Hypoxia treatment

Hypoxia treatment (3.03-kPa oxygen) for *pPCO1:GFP* expression analysis in the *bdg1* mutant was performed on plants grown on standard ½ murashige and skoog (MS) medium in a gloveless anaerobic chamber (COY) for 12 h. The desired oxygen level was achieved by mixing nitrogen, oxygen, and atmospheric air.

### Dex treatment

Dex treatment was performed to induce *CDEF1* expression. Dex stock solution was prepared by dissolving Dex in 96% ethanol and then diluting it in water for treatment, with control plants treated with an equivalent amount of ethanol diluted in water without Dex. Young seedlings (10 days old) were grown for 8 days on standard MS medium with 0.5% sucrose before being transferred to the same medium containing 10 μM Dex. Imaging or TB staining was performed 48 h after treatment. Older plants (4 weeks old) were sprayed once with 30 μM Dex before performing oxygen measurements. Control plants for both age groups were treated with ethanol solution matching the volume used for Dex treatments.

### Meristem dissection

To perform oxygen measurements, X-ray μCT scanning, and microscopy visualizations, the vegetative meristems of 4-week-old or 3- to 4-day-old *Arabidopsis* or 10-day-old Micro-Tom plants were dissected using injection needles, razor blades, tweezers and a Leica binocular stereo microscope.

### Vibratome longitudinal sectioning

To better visualize the SAMs of 10-day-old or 4-week-old plants with leaves covering the meristem, longitudinal vibratome sectioning was performed. Prior to sectioning, samples were fixed in 4% paraformaldehyde for 1 h at room temperature and then washed in 1× PBS solution (137 mM NaCl, 2.7 mM KCl, 10 mM Na_2_HPO_4_, and 2 mM KH_2_PO_4_, pH 7.4). The plants were positioned on a plastic lid for an Eppendorf-like tube with the bottom removed. For longitudinal sectioning, the samples were positioned sideways. The tube was then used to enclose the sample, and 4% agarose gel was poured into the tube over the plant. The tube with agarose and the plant was placed in an ice bucket until the agarose solidified. Once solid, the lid was removed, and the agarose-embedded sample was pushed out of the tube. The agarose plug was cut to approximately 1 cm in height using a knife. The agarose block was then glued to the vibratome disk using super glue. The disk with the attached agar block was assembled onto the vibratome (VT1000S), and longitudinal sections were cut with the following settings: speed 9, frequency 7, and section thickness 120 μm. Sections were carefully transferred using a brush into 1× PBS medium and imaged using either a Leica M205 FCA widefield microscope or a Zeiss Axio Imager 2 widefield fluorescence microscope.

### Microprofiling of oxygen in the SAM

Oxygen profiling was performed on the SAMs of 4-week-old *Arabidopsis* plants, 10-day-old Micro-Tom seedlings, and *Arabidopsis* inflorescences using a custom-built Clark-type microsensor with 3- to 5- and 10-μm-thick tips (Unisense). This microsensor was connected to a pA meter (Unisense) and attached to a motorized micromanipulator (MM33, Unisense). The microsensor tip step size was 10 μm for *Arabidopsis* and 30 μm for Micro-Tom, starting from outside the tissue and penetrating it until the target tissue was fully accessed. Prior to measurements, the microsensor was calibrated under two conditions: 0-kPa oxygen using ascorbic acid solution (50 ml of sodium ascorbate in 50 ml of 0.1 M NaOH) and 21-kPa oxygen using oxygenated medium (bubbled with air prior to calibration). Accurate positioning over the target tissue was performed by using a boom-stand dissection microscope (Zeiss Stemi 305). The resistance of the meristem surface to the microsensor occasionally resulted in a slight pushing of the tissue rather than direct penetration. This was taken into account in the representation of the oxygen profiles and indicated by a dashed line, indicated technical uncertainty. All measurements were performed under controlled conditions at 20°C in dim light.

### SAM height and width

SAM height and width were measured using microscopy pictures of longitudinal vibratome sections taken with a 40× objective from cuticle biosynthesis mutants, cutinase-overexpressing plants, and WT. Height was determined by drawing a horizontal line connecting the visible larger leaf primordia, which marked the boundary at the start of the large, oval, potentially differentiated cells. A perpendicular line was then drawn from the top of the SAM to this horizontal line and measured. Width was measured by drawing a line between the two youngest leaf primordia.

### Microscopy

To detect *pPCO1:GUS-GFP* fluorescence in 3- to 10-day-old *Arabidopsis* seedlings, 4-week-old vegetative meristems, and IMs, plants were fixed in 4% paraformaldehyde for 1 h at room temperature and then washed in 1× PBS. Meristems were dissected using a vibratome (VT1000S) into 120-μm sections and imaged using a Zeiss Axio Imager 2 widefield fluorescence microscope with differential interference contrast (DIC) function and a GFP filter (excitation 488 nm, emission 509 nm), an LSM Zeiss 700 AxioObserver (eGFP excitation 488 nm, emission 523 nm; chlorophyll excitation 639 nm, emission 660 nm), or an LSM Zeiss Airyscan 800 (eGFP excitation 488 nm, detection 410–547 nm; FM4-64 excitation 506 nm, detection 547–640 nm). Fluorol Yellow staining was imaged using the Zeiss Airyscan 800 with excitation at 488 nm and detection between 410 and 575 nm. Imaging of cuticle GFP reporter lines was performed using the Zeiss Airyscan 800 (eGFP excitation 488 nm, detection 410–547 nm; FM4-64 membrane staining excitation 506 nm, detection 547–640 nm). TB staining was imaged using a Leica M205 FCA widefield fluorescence microscope. mTurquoise fluorescence in the SAM of 4-week-old plants was imaged using a Leica M205 FCA widefield fluorescence microscope with a GFP filter, and for 10-day-old plants using the Zeiss Axio Imager 2 widefield microscope (excitation 400–460 nm, emission 500–600 nm). Differential contrast images were acquired using the Zeiss Axio Imager 2 microscope. GUS staining was imaged using a Leica THUNDER Imager fluorescence microscope. Dissected Micro-Tom SAMs were imaged using a Leica M205 FCA widefield microscope. Confocal laser scanning microscopy for EdU staining was performed using a Zeiss LSM 900 (excitation 590 nm, emission 573–700 nm). Imaging of FM4-64-stained Micro-Tom SAMs for volume measurements was done using a Zeiss LSM 900 (excitation 506 nm, detection 547–640 nm). *pHRPEx5:UnaG-mCherry* plants were imaged using the Zeiss Axio Imager 2 widefield microscope (UnaG excitation 450–490 nm, emission 500–550 nm; mCherry excitation 538–562 nm, emission 570–640 nm).

### Mean GFP and GUS signal intensity quantification

GFP or GUS raw leica image file (LIF) microscope images were processed in Fiji/ImageJ. Images were converted to 32-bit, a fixed intensity threshold was applied to isolate the signal from the noise, and background pixels were set to not a number (NaN). The same threshold value was used for all images. ROIs were drawn manually with the polygon tool to select the SAM and the two youngest leaf primordia. Mean fluorescence or GUS gray pixel intensity was quantified using Fiji/ImageJ.

### Gene expression analysis

2-week-old *pML1:GR-LHG4:pOp4:CDEF1* plants were sprayed with 30 μM Dex solution (water) and ethanol solution (control: the same amount of 96% ethanol was added to water). After 2 or 4 days, the apices (three per sample) were collected and RNA was extracted using QIAGEN RNeasy-kit. DNAse I digestion was used to remove genomic DNA. Following RNA extraction, complementary DNA (cDNA) was synthesized using the RevertAid First Strand cDNA Synthesis Kit (Thermo Fisher Scientific). RT–qPCR was performed using SYBR Green Real-Time Master Mix with 10 ng of cDNA per reaction. The primers used in this study are listed in Supplemental Table 5.

### Sugar starvation via prolonged dark treatment

*Arabidopsis* Col-0 plants were grown under short-day conditions with a 9-h light period (Z0–Z9 [Zeitgeber time]) followed by 15 h of darkness (Z9–Z24). For the first sample, oxygen measurements in the SAM were conducted closer to dawn at Z24, starting from Z22.5 to Z0 (lights on). These plants are expected to be at the limits of their sugar-storage reserves. For the control sample, oxygen measurements were performed during the middle of the light period, 5–6 h after lights on (Z5–Z6). Finally, the extended night sample was measured after 20 h of darkness, from Z20 (5–6 h after the expected lights on) to Z21.

### Cell proliferation detection by EdU

The dissected Micro-Tom vegetative meristems were incubated in 10 μM EdU (Invitrogen) solution for 2–3 h in 1.5-ml PCR tubes. After incubation, the EdU solution was removed and replaced with 90% acetone, and the tubes were placed on ice for 10 min. Following fixation, the tissues were given three washes with 1× PBS and fixed in FAA (10 ml of formaldehyde [37%–40%], 5 ml of glacial acetic acid, 50 ml of ethanol, and 35 ml of H_2_O) for 2–3 h. The meristems were washed twice with 0.5% Triton X-100 in 1× PBS. The reaction cocktail (Invitrogen) containing Alexa Fluor 594 was added to the tubes, and the meristems were incubated for 30 min in the dark. Afterward, the tissues were washed three times with 1× PBS. EdU signals were observed using a Zeiss LSM900 confocal microscope with a water-dipping 40×objective .

### Respiration rate measurements by the Unisense nanorespiration system

For the experiment comparing respiration rates after growth on 2% vs. 0% sucrose, 10-day-old Micro-Tom seedlings were dissected to remove cotyledons, leaves, and hypocotyl. Samples approximately 600 μm in length (including the SAM [∼200 μm] and part of the subapical region that facilitates sucrose uptake) were incubated for 24 h on medium containing 1× MS powder, 2% or 0% sucrose, 1.5% agarose, pH 5.8, gibberellic acid A3 (0.01 μM, Sigma), kinetin (0.01 μM, Sigma), and plant preservative mixture (PPM™) (1 μl/ml, Plant Cell Tech) before use for respiration measurements.

For modeling, only samples approximately 250 μm in length, including the meristem (∼200 μm) and a small portion of the hypocotyl (∼50μm), were dissected from 10-day-old Micro-Tom seedlings to match the size of μCT-scanned meristems and ensure that respiration was measured specifically from the meristematic region.

The nanorespiration system consisted of a Clark-type oxygen microsensor (OX10; tip diameter 10μm) mounted on a motorized micromanipulator (MM33), attached to a laboratory stand (LS18), and connected to an amplifier (fx-6 UniAmp) for signal recording and a nanorespiration-measuring unit. The measuring unit included seven fused glass capillaries (inner diameter 0.68 mm, height 3 mm) with sealed glass bottoms, arranged in a rosette disk. The rosette was fitted into a disk holder mounted on the laboratory stand using a metal frame.

Capillaries were filled with medium (the same medium that was used to grow the SAMs but liquid), and individual dissected SAMs were placed at the bottom of five capillary wells with one or two capillaries left without a sample for control measurements. The samples were equilibrated with atmospheric oxygen for 30 min prior to the measurements. Oxygen concentration at the air–liquid interface was assumed to be in equilibrium with ambient air, as confirmed by control vials without meristems, which showed stable oxygen levels. The oxygen microsensor was calibrated during this time in 0-kPa oxygen using ascorbic acid solution (50 ml of sodium ascorbate in 50 ml of 0.1 M NaOH) and in 21-kPa oxygen using oxygenated medium (bubbled with air prior to calibration). The sensor was manually positioned in the wells of the glass capillaries using a stereo microscope (Zeiss Stemi 305 with boom stand). Measurements were taken stepwise, with step sizes of 200 μm, up to a depth of 1000–1400 μm to form an oxygen profile. All measurements were performed under controlled conditions at 20–21°C under dim light.

The respiration rate was determined by analyzing the linear oxygen concentration gradients generated by SAM respiration within the capillaries. The oxygen flux was calculated using Fick’s First Law of Diffusion:

Oxygenflux(nmolms−1−2)=−DΔCΔx

where D is the diffusion coefficient of oxygen in the medium (approximately 2.1 × 10^−9^ m^2^ s^−1^ at 20°C) and *dC/dx* represents the measured oxygen concentration gradient (the slope). The cross-sectional area of the capillary was calculated based on its inner diameter:

Cross−sectionalarea=π×(6.8∗10−6m2)2≈3.63×10m2−11

The oxygen consumption or respiration rate was then determined by multiplying the oxygen flux by the cross-sectional area:

$$\text{Respiration}\;\text{rate}\;\left(\text{nmol}\;s^{-1}\;\text{or}\;\text{nmol}\;h^{-1}\right)=\text{Oxygen}\;\text{flux}\times \text{Cross}-\text{sectional}\;\text{area}$$

### Chemical inhibition of respiratory activity in the SAM

To assess how respiratory activity affects oxygen uptake in the SAM, the cytochrome *c* pathway was inhibited with 2 mM KCN and the AOX pathway with 10 mM salicylhydroxamic acid (SHAM). The concentration of the chemicals was determined from studies that applied these inhibitors to leaves, and the duration of treatment was optimized experimentally (Bartoli et al., 2006). Dissected meristems were first grown for 24 h on medium (1× MS powder, 2% sucrose, 1.5% agarose, pH 5.8, gibberellic acid A_3_, kinetin, and PPM [1 μl/ml]) to recover after dissection. SAMs were incubated for 30 min in the same medium without agarose and containing the respiratory inhibitors under dim light. Before oxygen measurements, the SAMs were rinsed with inhibitor-free medium.

### Volume measurements

Optical sectioning with a water-dipping objective was used to quantify the volume of meristems used for respiration measurements and modeling. Confocal z stacks of Micro-Tom vegetative meristems (250 μm) with FM4-64 membrane staining were analyzed in MorphoGraphX (MGX). Stacks were preprocessed by applying a Gaussian blur (σ = 0.3 μm) on all axes to reduce noise. The Edge Detect algorithm was then used to create a solid structure based on the global shape of each individual meristem (threshold = 10 000, multiplier = 2.0, adapt factor = 0.3, fill value = 30 000). If needed, gaps in the tridimensional structure were closed by multiple passes of the Fill Holes function (*x*,*y* radius = 10, threshold = 10 000, depth = 0, fill value = 30 000). Regions adjacent to the meristematic dome were manually excluded from the structure for further analysis. A 3D surface mesh encompassing each meristem was created via the Marching Cube Surface algorithm (cube size = 5 μm, threshold = 5000). The mesh was then refined via the Subdivide and Smooth Mesh (20 smoothing passes) processes, respectively. The resulting tridimensional structure was identified as a single object via watershed segmentation, and the volume was computed via the Heatmap/Measures3D/Geometry/Volume function. Finally, the 3D reconstruction of the meristems was completed by projecting the surface fluorescent signal on the mesh via the Project Signal function (minimum distance = 2 μm, maximum distance = 6 μm, minimum signal = 0, maximum signal = 60 000).

### X-ray μCT

X-ray μCT scanning was performed on a vegetative meristem of Micro-Tom tomato (*S. lycopersicum* L.) at 9–10 days after germination and the 7-day-old seedling and IM of *A. thaliana*. The excised vegetative meristem of tomato and the IM of *A. thaliana* were covered in parafilm to prevent dehydration during scanning. The *A. thaliana* seedling was enclosed between two pieces of Kapton tape and secured with parafilm in a pipette tip for mounting in the sample holder. X-ray projection images of the meristem sample were obtained using a UniTom HR μCT system (Tescan XRE nv, Ghent, Belgium) at 75 kV and 1 W. In total, 1900 images with a voxel resolution of 855 nm were captured per sample. With an exposure time of 1100 ms, this resulted in a total scan time of 45 min for each meristem. To obtain the 3D volumetric image of the meristem, the projections were reconstructed using the filtered back-projection method in Panthera (Tescan XRE nv, Ghent, Belgium), and spot and ring filters were applied to improve image quality.

### X-ray image processing

The reconstructed 16-bit gray-scale image stack containing the shoot apex (SAM, subapical zone, and part of the hypocotyl) and surrounding leaves was converted to 8-bit using MATLAB R2020b (The Mathworks, Natick, MA, USA). Subsequent image segmentation was conducted in Avizo 2021.3 (Thermo Fisher Scientific, Waltham, USA). First, image noise was reduced by median filtering. The parafilm wrappings visible in the image were masked out. A region of interest (ROI) was selected that covered the SAM and subapical zone with leaf primordia and a part of the hypocotyl. A 3D geometric surface model of the apex, separating the individual tissues using interpolation operations, was then constructed from the masked ROI and saved as an .stl file. To reduce geometric and computational complexity, trichomes were ignored and morphological corrections were applied to obtain a smooth surface model. The surface model was filled with a finite-element tetrahedral mesh for establishing and solving the 3D reaction-diffusion model of oxygen transport, using the mesh generator of Comsol Multiphysics 6.0 (Comsol, Stockholm, Sweden).

Otsu’s thresholding (Otsu, 1979) was used to segment the intercellular air spaces from the meristem tissue on the reconstructed filtered μCT images. The tissue porosity was calculated as the volume of pore spaces to the volume of the whole tissue. Effective oxygen diffusivity was calculated based on tissue porosity (Nugraha et al., 2021). If no pores were evident from the μCT image data, porosity was assumed to be zero, and an approximate oxygen diffusivity value was used (effective oxygen diffusivity in water at 20°C: 5 × 10^−11^ m^2^ s^−1^).

### Reaction-diffusion modeling of oxygen transport in meristems

To simulate the oxygen levels inside the meristem, a reaction-diffusion model for oxygen transport (Ho et al., 2008) was applied accounting for diffusion and respiration at atmospheric conditions (21 kPa at 21°C). Isotropic effective diffusivity and porosity values were assigned to the different tissues based on the μCT scan analysis. The permeability of the cuticle was set to 1 × 10^−7^ m s^−1^, which was based on published values for multiple plant species (Lendzian, 1982; Lendzian and Kerstiens, 1991; Zabalza et al., 2009). Respiratory oxygen consumption of the tissues was modeled using a Michaelis–Menten model. The maximal consumption rate was taken from respiration measurements of excised apex (SAM and subapical zone) using oxygen sensors (1 × 10^−2^ mol m^−3^ s^−1^ for the apex), while a maximal consumption rate of 1 × 10^−3^ mol m^−3^ s^−1^ was assumed for the hypocotyl. The Michaelis–Menten constant *Km* value was set equal to the value reported for mitochondrial cytochrome *c* oxidase (0.1 μM or 1 × 10^−2^ kPa). Photosynthetic production of oxygen was neglected. Boundary conditions were a permeation flux across the cuticle at 21-kPa external oxygen partial pressure and a constant level of 21 kPa at the basal hypocotyl surface, which was assumed to be well oxygenated. The 3D reaction-diffusion model was solved using Comsol Multiphysics 6.0 (Comsol AB, Stockholm, Sweden). The sensitivity of the oxygen distribution to cuticle permeability, respiration, and diffusivity due to porosity was evaluated through parameter sweep simulations. All parameters are listed in Supplemental Table 1.

To simulate the tissue-specific respiration in the SAM, the reaction-diffusion model for oxygen transport was modified to consider the heterogeneous nature of the SAM. The model geometry was adapted to create subdomains for the CZ, OC, PZ, and primordia (Supplemental Figure 13). The delineation and volume of these tissues were based on literature (Weits et al., 2019). Respiration rates of individual tissues are currently technically not possible. To test how heterogenous metabolism in the SAM would affect internal oxygen distribution, we therefore assumed in our model that the respiration rate of the tissue region subdivisions would be proportional to the division rates in the tissue region. Based Reddy et al. (2004) and Kitagawa and Jackson (2019), the average division time was assumed to be equal to 54 h for the CZ, 27 h for the PZ, and 15 h for the primordia. The cell-division duration of the OC was assumed to be two times longer than for the CZ and was set to 108 h. The respiration rate of each tissue zone was scaled by the inverse of the relative cell division time:

(Equation 1) $$R_{\text{CZ}}=0.33\;R_{primordia}$$

(Equation 2) $$R_{\text{OC}}=0.14\;R_{primordia}$$

(Equation 3) $$R_{\text{PZ}}=0.5\;R_{primordia}$$

The respiration of the apex is equal to the sum of respiration of the tissue regions. Consequently, the respiration rate of the apex is equal to the respiration rate of the tissue regions weighted by their respective volume fraction (x):

(Equation 4) $$R_{\text{apex}}=R_{\text{cz}}X_{\text{Cz}}+R_{\text{oc}}X_{\text{OC}}+R_{\text{pz}}X_{\text{pz}}+R_{primordia}X_{primordia}$$

Solving Equation 4 by substituting the respiration rates as per Equations 1, 2, and 3 and volume fractions from Supplemental Table 2 yields the respiration rates summarized in Supplemental Table 3.

### Statistical analysis

Statistical analysis was performed with GraphPad Prism 10 or R version 3.6.1 using the indicated statistical tests, and differences were significant at *p* < 0.05.

## Funding

The authors acknowledge the funding provided by 10.13039/501100000781European Research Council (ERC-LOKI: 101077812) and 10.13039/501100003246De Nederlandse Organisatie voor Wetenschappelijk Onderzoek (NWO-Vidi, MorphO2gen: VI.Vidi.213.055), Research Foundation Flanders – FWO Vlaanderen (B.D., FR scholarship no. 1189422N; L.V.D., SBO FoodPhase grant number S003421N). The financial support of 10.13039/501100003130FWO (grant number I013518N) and 10.13039/501100004040KU Leuven (project C1 C14/22/076) for the XCT Core Facility is gratefully acknowledged.

## Acknowledgments

We thank the NPEC facility manager, Dr. Valerian Meline, for performing the phenotyping of cuticle biosynthesis mutants. We thank Assistant Professor Dr. Marcel Proveniers and Dr. Livia Merendino for kindly providing the *clv3-15* and *rpoTmp/aox1a* seeds. No conflict of interest is declared.

## Author contributions

D.A.W. and V.V. conceptualized the project and wrote the manuscript. D.A.W., V.V., B.D., and L.V.D. conducted the experiments. G.P. performed volume measurements of the SAM and gene expression analysis. B.D., L.V.D., P.V., and B.N. simulated oxygen transport using the reaction-diffusion model. B.D., L.V.D., P.V., P.P., and B.N. edited the manuscript. All authors have read and agreed to the published version of the manuscript.

## Supplemental information

Document S1. Supplemental Figures 1–13 and Supplemental Tables 1–5

Document S2. Article plus supplemental information
