# Supplementary material for: The hypoxic niche enclosing the shoot apical meristem is shaped by a combination of morphological features and metabolic activity
Source: Mol Plant. 2026 May 4;19(5):1080–99. doi: 10.1016/j.molp.2026.02.011 (PMC13139043; doi:10.1016/j.molp.2026.02.011)
Supplement: Document S1. Supplemental Figures 1–13 and Supplemental Tables 1–5 [file mmc1.pdf]

Molecular Plant, Volume *19*

Supplemental information

The hypoxic niche enclosing the shoot apical meristem is shaped by a combination of morphological features and metabolic activity

Viktoriia Voloboeva, Bart Dequeker, Leen Van Doorselaer, Gabriele Panicucci, Pierdomenico Perata, Pieter Verboven, Bart Nicolai, and Daan A. Weits

Supplemental information for

The hypoxic niche enclosing the shoot apical meristem is shaped by a combination of morphological features and metabolic activity

Viktoriia Voloboeva[1,2,3], Bart Dequeker[4], Leen Van Doorselaer[4], Gabriele Panicucci[1], Pierdomenico Perata[2], Pieter Verboven[4], Bart Nicolai[4,5], Daan A. Weits[1].

[1]Experimental and Computational Plant Development, Institute of Environment Biology, Utrecht University, Padualaan 8, Utrecht, 3584 CH the Netherlands

[2]PlantLab, Institute of Plant Sciences, Scuola Superiore Sant'Anna, 56010 Pisa, Italy

[3]National Enterprise for nanoScience and nanotechnology, 56010 Pisa, Italy

[4] Division of Mechatronics, Biostatistics and Sensors (MeBioS), Department of Biosystems, KU Leuven, Willem de Croylaan 42, 3001 Leuven, Belgium

[5]Flanders Centre of Postharvest Technology, Willem de Croylaan 42, 3001 Leuven, Belgium

Corresponding author: Daan A. Weits

Email: d.a.weits@uu.nl

Supplemental table 1. Parameters used in the reaction-diffusion model

Model parameter	Value	Unit	Source
Cuticle permeability			
$h_{cuticle}$	1 * 10^{-7}	m s^{-1}	Literature (MacFarlane, 1992; Frost-Christensen et al., 2003): leaf cuticle of submerged aquatic plants down to 1 x 10^{-6} m s^{-1}
Respiration rate			
$R_{oxygen\ experiment}$	0.5 ± 0.154	nmol h^{-1} per meristem	Experimentally measured respiration rate of 250 µm SAM (n=14)
$V_{sample\ size}$	0.017 ± 0.0019	mm^3	Volume measured using confocal z-stacks and MorphoGraphX (n=6)
$R_{oxygen\ in\ apex}$	1 * 10^{-2}	mol m^{-3} s^{-1}	Volumetric maximal oxygen consumption rate
$R_{oxygen\ in\ hypocotyl}$	1 * 10^{-3}	mol m^{-3} s^{-1}	Assumption: lower respiration rate below apex
K_M	1 * 10^{-7}	mol m^{-3}	Literature (Zabalza et al., 2009): K_M of cytochrome c oxidase
Diffusivity			
$D_{oxygen\ in\ shoot\ apex}$	5 * 10^{-11}	m^2 s^{-1}	Effective gas diffusivity of water (based on CT images: very low porosity & very low connectivity in SAM)
$D_{oxygen\ in\ hypocotyl}$	10 * 10^{-11}	m^2 s^{-1}	Effective gas diffusivity in hypocotyl (based on CT images)

Parameters are grouped into three categories: cuticle permeability, respiration rate, and diffusivity. The source specifies whether parameters were measured in this study or derived from published literature.

Supplemental table 2. Volumes and volume fractions of the tissue regions. CZ: central zone. OC: organizing centre. PZ: peripheral zone.

Tissue	Volume (µm^3)	Volume fraction (%)
Primordia	9.09 x 10^7	92.27
CZ	3.62 x 10^5	0.37
OC	1.68 x 10^5	0.17
PZ	7.09 x 10^6	7.20

Supplemental table 3. Parameters for the reaction-diffusion model accounting for tissue-specific respiration in the SAM. CZ: central zone. OC: organizing centre. PZ: peripheral zone.

Model parameter	Value	Unit	Source
Cuticle permeability			
$h_{cuticle}$	1 x 10^{-7}	m s^{-1}	Literature (MacFarlane, 1992; Frost-Christensen et al., 2003): leaf cuticle of submerged aquatic plants down to 1 x 10^{-6} m s^{-1}
Respiration rate			
$R_{hypocotyl}$	1 x 10^{-3}	mol m^{-3} s^{-1}	Assumption: lower respiration rate below apex
R_{apex}	1 x 10^{-2}	mol m^{-3} s^{-1}	Experimentally measured respiration rate of 250 µm of excised SAM (n=14)
R_{CZ}	3.44 x 10^{-3}	mol m^{-3} s^{-1}	Respiration rate scaled from R_{apex} by relative cell division time (Reddy et al., 2004; Kitagawa et al., 2019)
R_{OC}	1.46 x 10^{-3}	mol m^{-3} s^{-1}	
R_{PZ}	5.21 x 10^{-3}	mol m^{-3} s^{-1}	
$R_{primordia}$	1.04 x 10^{-2}	mol m^{-3} s^{-1}	
K_M	1 x 10^{-7}	mol m^{-3}	Literature (Zabalza et al., 2009): K_M of cytochrome c oxidase

Diffusivity			
$D_{oxygen\ in\ shoot\ apex}$	5×10^{-11}	$m^2\ s^{-1}$	Effective gas diffusivity of water (based on CT images: very low porosity & very low connectivity in SAM)
$D_{oxygen\ in\ hypocotyl}$	10×10^{-11}	$m^2\ s^{-1}$	Effective gas diffusivity in hypocotyl (based on CT images)

Supplemental table 4. Genotyping primers and PCR conditions for T-DNA insertion screening

Line	Mutant	Target gene	FW (5' to 3')	RV (5' to 3')	Notes
SAIL_1286_E08	*bdg1*	AT1G64670	GTGGAAACCCTGCTACTGCT	AGGACAACGAAACAGGGGAC	FW+LB 58 °C
WiscDsLox245B03	*dcr1-3*	AT5G23940	GTGGAAACCCTGCTACTGCT	AAGTCGTGTATAGTGTTTTGTGATT	FW+LB 56 °C
SALK_150886C	*gpat4*	AT1G01610	GTCAGTTGAGCGATAAACAACCA	AAGATTAAAAATCGAAAGGGTTCCA	RV+LB 51 °C
SALK_095122	*gpat8*	AT4G00400	GCCCTACACTTTACGCTCTTAGT	GAACACCAGGCTTCTTCACA	FW+LB 55 °C
Insertion	Primer sequence				
SAIL LB (*bdg1* genotyping)	TAGCATCTGAATTTCATAACCAATCTCGATACAC				
P745 LB (*dcr1-3* genotyping)	AACGTCCGCAATGTGTTATTAAGTTGTC				
SALK LBb1.3 (*gpat4* genotyping)	ATTTTGCCGATTTCGGAAC				
SALK LBb1 (*gpat8* genotyping)	AACCAGCGTGGACCGCTTGCTG				

The table shows genomic (FW and RV) and left border (LB) primers, recommended primer combinations for T-DNA identification, and corresponding annealing temperatures. Sequences are shown from 5' to 3'.

Supplemental table 5. Cloning and qPCR primers

Primer name	Primer sequence (5' to 3')
gg_ML1_Fw	aacaGGTCTCaACCTGACGAAAGGGCCTCG
gg_ML1_Rv	aacaGGTCTCaTGTTTCGCGCGTTTCGGTG
gg_CDEF1_Fw	aacaGGTCTCaGGCTccATGGTCGAGGGAGAG
gg_CDEF1_Rv	aacaGGTCTCaCTGATTCAGTAGCACGTCTG
gg_pop6_Fw	aacaGGTCTCaACCTATGCATATGTCGAG
gg_pop6_Rv	aacaGGTCTCaTGTTCGTCCTCTCCAAATG
qpcr_HEC1_Fw	GGGAGTCGTTATGAAAGGGTGTGG
qpcr_HEC1_Rv	ATCTGTGCATTGCCCACCATCTG
qpcr_ATH1 _Fw	ACCTCAACGAGGTTTGCCTGAG

qpcr_ATH1 _Rv	TCTCCGAATCTTTCGGGTAAGGG
qpcr_BAM2 _Fw	ACGTGACTGAGAAAGCTCCG
qpcr_BAM2 _Rv	CAAACCCAATCGCCGGAAAG
qpcr_CLV3 _Fw	TAAGGACTGTTCCTTCGGGACCTG
qpcr_CLV3 _Rv	TCTTGGCTGTCTTGGTGGGTTC
qpcr_WUS _Fw	TCATCACGGTGTTCCCATGCAG
qpcr_WUS _Rv	CCCGTTATTGAAGCTGGGATATGG
qpcr_ZPR1_Fw	CATTTTCAGACACACCCACGA
qpcr_ZPR1_Rv	CTTTTTCTCTTCCCGCCACA
qpcr_REV_Fw	AACCACCGTGAGAGAAGCAG
qpcr_REV_Rv	TTGTTGTCGACGGAGAGAGC
qpcr_STM_Fw	ACCTTCCTCTTTCTCCGGTTATGG
qpcr_STM_Rv	GCGCAAGAGCTGTCCTTTAAGC
qpcr_CDEF1_Fw	ATGGTCGAGGGAGAGTCCAAGGC
qpcr_CDEF1_Rv	GACGACGAGCTAGAGTAGGAGGCG
qpcr_ACTIN_Fw	GGCGATGAAGCTCAATCCAAA
qpcr_ACTIN _Rv	GGTCACGACCAGCAAGATCAAGACG

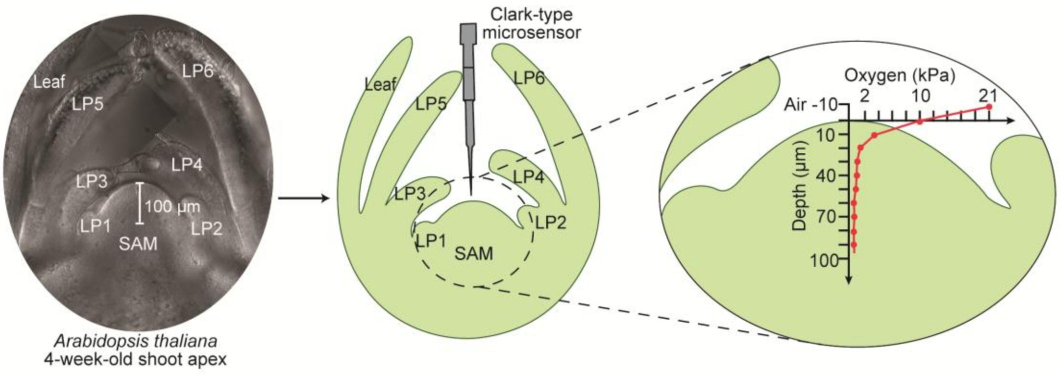

Supplemental figure 1. Clark-type microsensor oxygen profiling of the shoot apical meristem

(A) Schematic representation of oxygen level measurements using a Clark-type oxygen microsensor. The illustration shows the actual spatial resolution of the sensor as it penetrates the shoot apical meristem (SAM) tissue in vertical direction. The drawing was based on a vibratome section of a 4-week-old shoot apex, and the example oxygen profile was taken from Figure 1B. LP - leaf primordium.

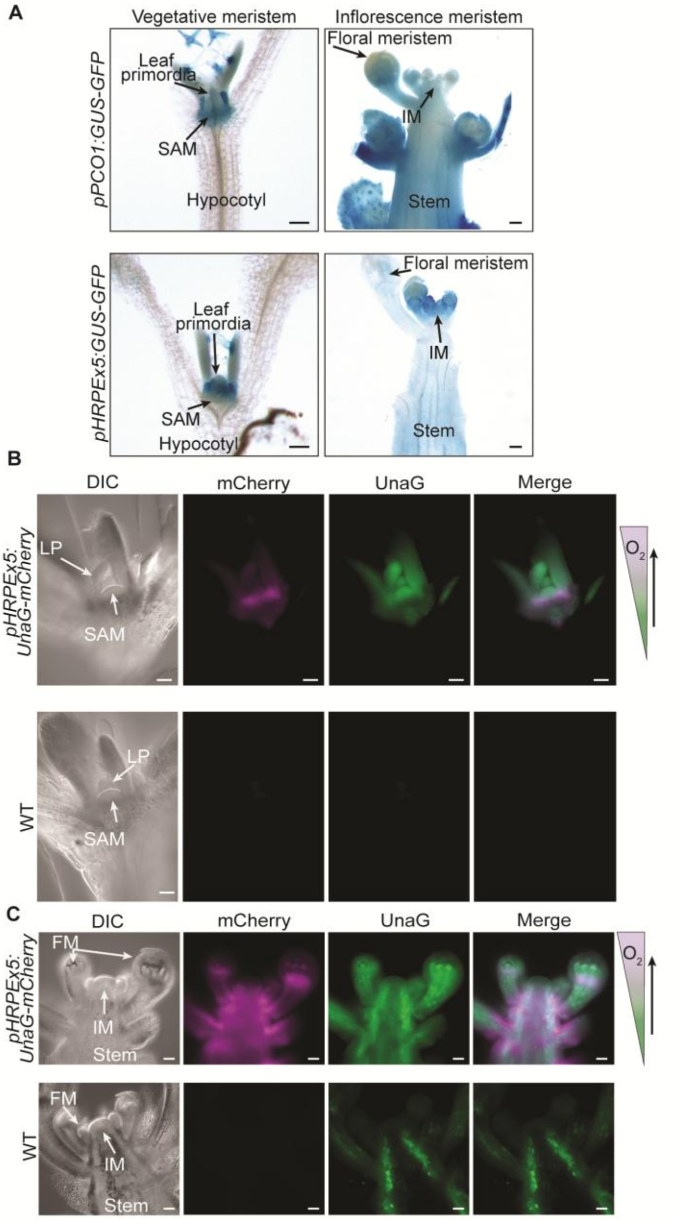

Supplemental figure 2. Alternative biosensors for oxygen levels and hypoxia signaling visualization in the SAM

(A) Comparison of GUS expression driven by *PCO1* and *HRPEx5* promoters to assess hypoxia distribution in the vegetative and inflorescence meristems (IM) and the surrounding tissues (n=5-8). Scale bar 100 µm.

(B-C) Hypoxia and oxygen level visualization in the vegetative (**B**) and inflorescence meristem (**C**) of Arabidopsis using a hybrid transcriptional and maturation-based biosensor in which the *HRPEx5* promoter responds to hypoxia, whereas UnaG-mCherry provides a ratiometric function (n=12-15). In the pHRPEx5:UnaG-mCherry biosensor UnaG matures independently of oxygen, whereas mCherry requires oxygen for fluorescence (Panicucci et al., 2020). Leaf primordia - LP, floral meristem - FM. WT image was used to show autofluorescence signal. Scale bar 50 µm.

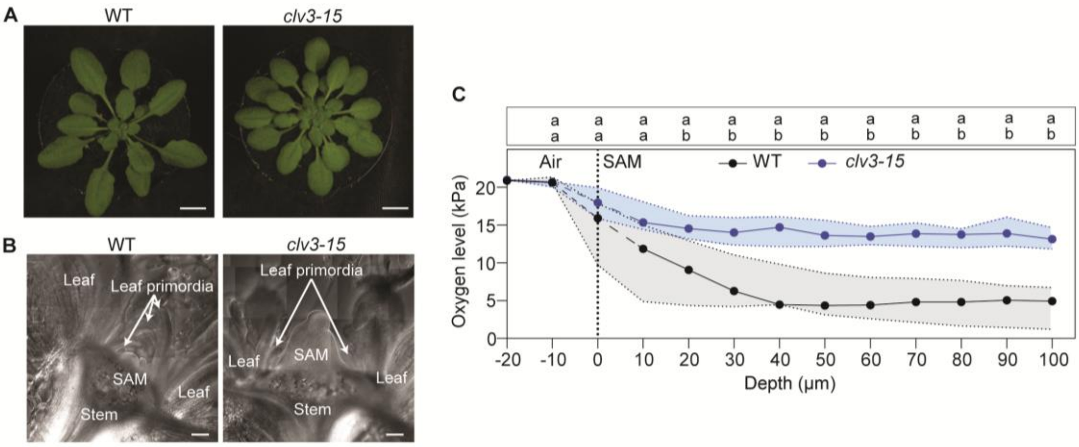

Supplemental figure 3. Effect of enlarged *clv3-15* SAM on oxygen levels

(A) Phenotype of 4-week-old *clv3-15* and WT plants used for oxygen measurements. Scale bar 1 cm (n=9). Pictures were taken with a Nikon Coolpix P520 digital camera.

(B) DIC images of SAMs from 3-week-old *clv3-15* and WT plants. The gray curve indicates the SAM area. Scale bar 100 µm.

(C) Oxygen measurements in the SAMs of 3- and 4-week-old *clv3-15* compared to WT plants performed using a Clark-type oxygen microsensor with a 10 µm tip. The vertical stippled line denotes the estimated position of the SAM boundary using microscopy. The dashed segment of the oxygen microprofile indicates the transition of the sensor across this boundary, where oxygen levels are less certain. Statistical differences were evaluated using two-way repeated-measures ANOVA, followed by Šídák's multiple-comparisons test (at each depth), $p < 0.05$, (n=5-6).

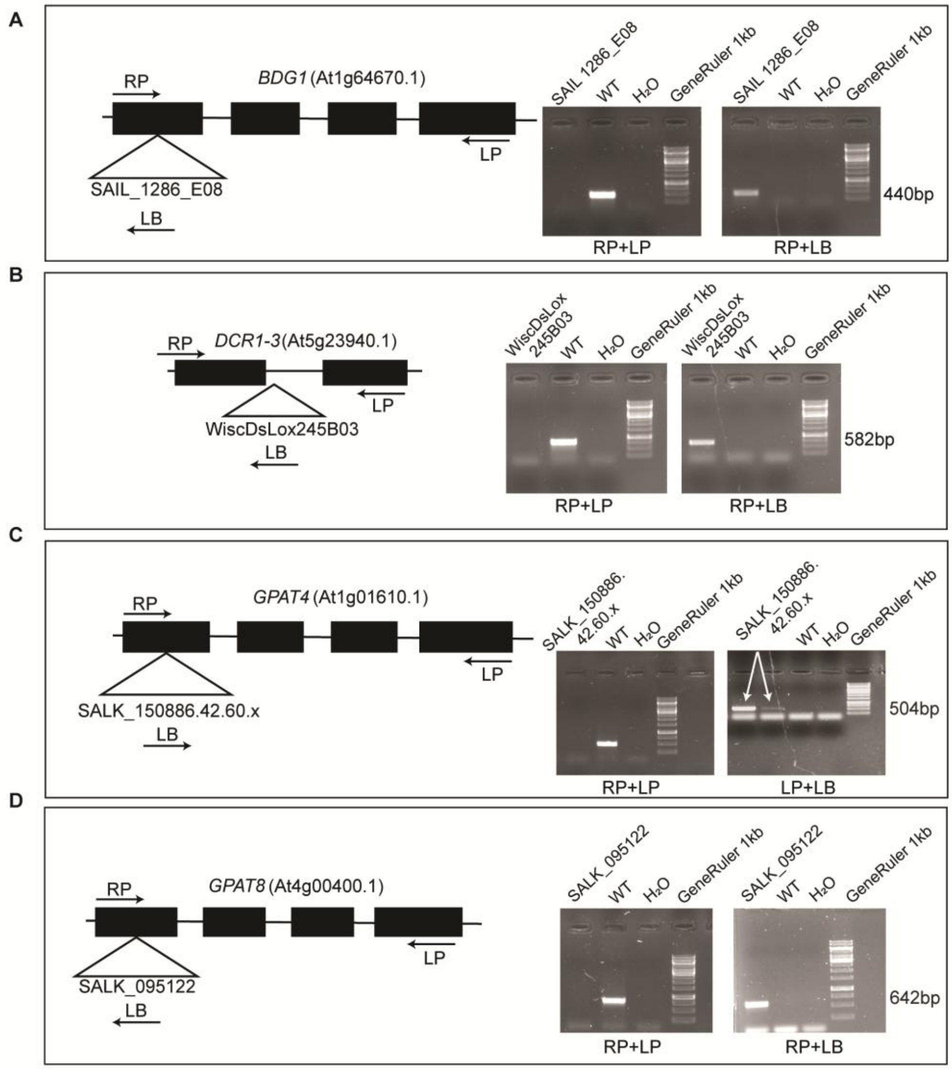

Supplemental figure 4. Identification of homozygous T-DNA insertion knockout lines.

(A-D) SAIL_1286_E08 for *BDG1* (At1g64670); WiscDsLox245B03 for *DCR1-3* (At5g23940); SALK_150886C for *GPAT4* (At1g01610); SALK_095122 for *GPAT8* (At4g00400). The left and right genomic primers (LP and RP) and the T-DNA border primer (LB) were used for validation of homozygous knockout lines. The gel photo shows that the LP-LB/RP-LB primer pairs were amplified, while the genomic primer pair LP-RP was not amplified (WT), confirming that the mutants were homozygous. The figure shows the location of T-DNA insertion in the gene. The arrow indicates the direction of a T-DNA insertion.

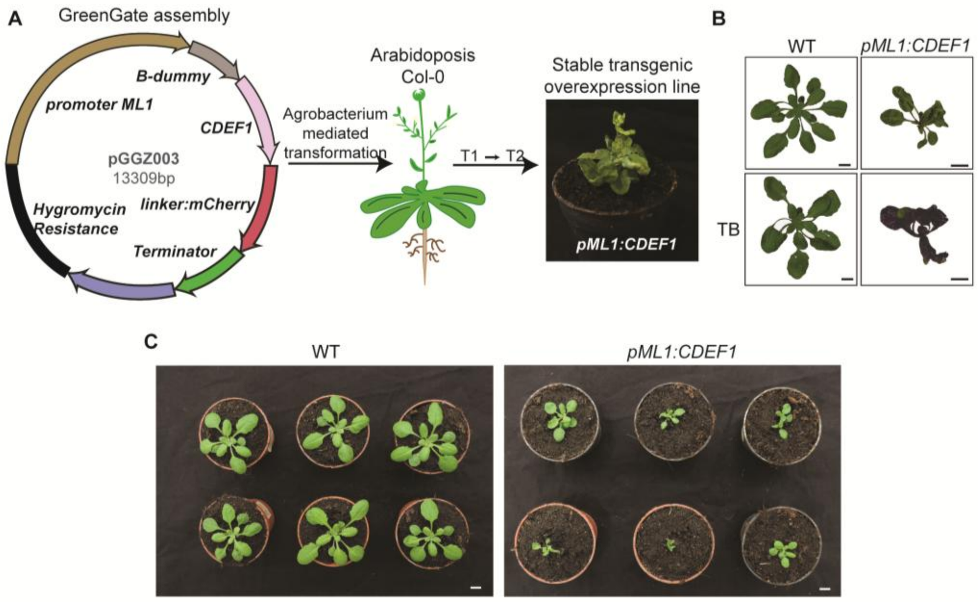

Supplemental figure 5. Phenotypic changes induced by cutinase overexpression.

(A) Scheme illustrating the Agrobacterium-mediated transformation of Col-0 Arabidopsis plants using a GreenGate-assembled construct containing the *CDEF1* coding sequence. The resulting transgenic plants, screened through T1 and T2 generations, exhibit a severe phenotype, potentially indicating cuticle defects.

(B) Toluidine blue staining showing permeable *pML1:CDEF1* plants in comparison to WT. The top images show the plants before staining, and the bottom images show them after staining. Scale bar 1 cm (n=5).

(C) Three-week-old *pML1:CDEF1* plants showing a variable phenotype compared to WT, reflecting the impact of cuticle defects on plant growth. Pictures were taken with a Nikon Coolpix P520 digital camera. Scale bar 1cm.

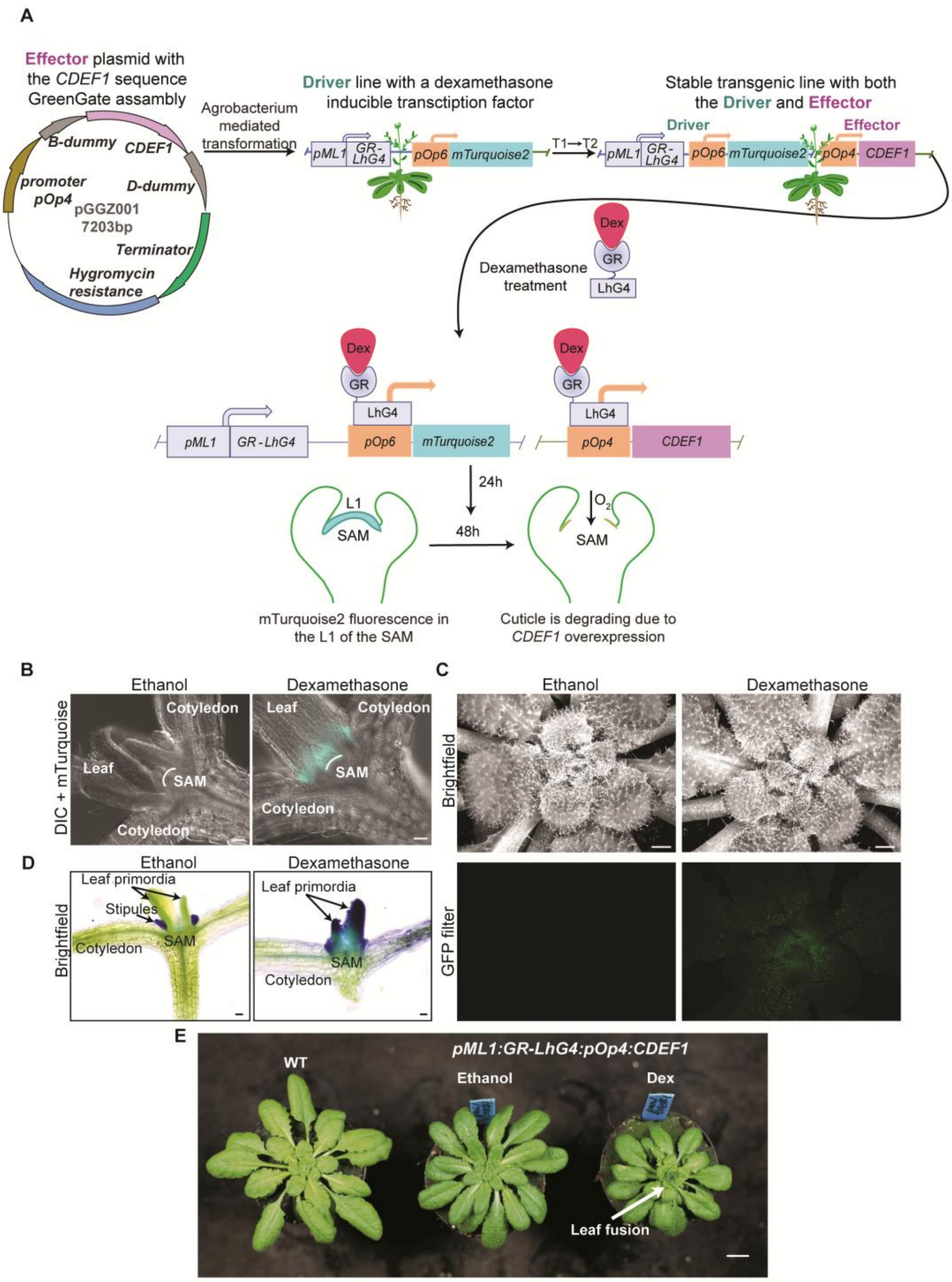

Supplemental figure 6. Cuticle removal via inducible CDEF1 overexpression.

(A) Schematic representation of the inducible, outermost meristem layer-specific CDEF1 overexpression system. The system consists of two components. The first is the effector, which we generated using the GreenGate cloning method allowing us to assemble the *pOp4* promoter with the CDEF1 coding sequence and a hygromycin resistance cassette. This effector construct was transformed via Agrobacterium into the second component, the driver line obtained from Schürholz et al. (2018) (Schürholz et al., 2018). The driver line contains a tissue-specific *pML1* promoter driving LhG4 fused to a rat glucocorticoid receptor (GR), along with the *pOp6* promoter containing LhG4 binding sites and an mTurquoise2 fluorescence reporter. The resulting transgenic plants, which contained both driver and effector components, could be treated with dexamethasone. This activates LhG4, which binds to the *pOp6*/*pOp4* promoter and drives the expression of both the mTurquoise2 fluorescent reporter and CDEF1. After approximately 24 hours (or less) of dexamethasone treatment, the mTurquoise2 signal is visible in the outermost layer of the SAM, where CDEF1 expression is induced, leading to cuticle degradation.

(B) mTurquoise2 visualization in the SAM vibratome sections of 10-day-old *pML1:GR-LHG4:pOp4:CDEF1* seedlings, showing fluorescence in the outermost layer. The images combine DIC and mTurquoise2 fluorescence. The left image shows an ethanol-treated plant with no signal, while the right image shows a dexamethasone-treated plant with a visible signal. Section thickness 120 µm. Scale bar 50 µm (n=6-8).

(C) mTurquoise2 visualization in the apices of 4-week-old *pML1:GR-LHG4:pOp4:CDEF1* plants treated with either dexamethasone or ethanol. Scale bar 2mm.

(D) Toluidine blue staining of SAM vibratome sections of 10-day-old *pML1:GR-LHG4:pOp4:CDEF1* plants, highlighting blue dye in the meristems and leaf primordia of dexamethasone-treated plants compared to ethanol-treated controls. Section thickness 120µm. Images were taken with a Leica M205 FCA stereo microscope. Scale bar 50 µm (n=6).

(E) Comparison of WT, ethanol-treated *pML1:GR-LHG4:pOp4:CDEF1* and dexamethasone-treated *pML1:GR-LHG4:pOp4:CDEF1* plants 10 days after spraying showing leaf fusion in the CDEF1-induced plants. Pictures were taken with a Nikon Coolpix P520 digital camera. Scale bar 1 cm.

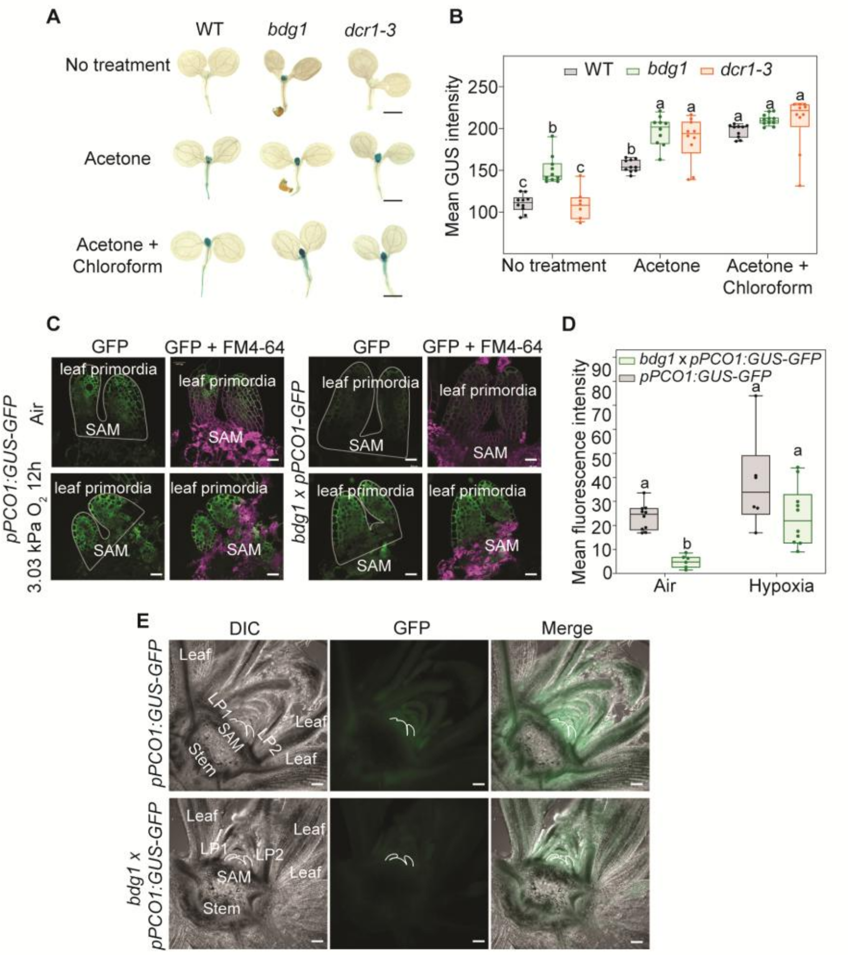

Supplemental figure 7. Cuticle defects and their effect on the response to hypoxia in the SAM.

(A) Images showing *pPCO1* expression in *bdg1, dcr1-3*, and WT 4-day-old seedlings located in the SAM and leaf primordia after three different treatments: 1) only GUS staining (No treatment), 2) acetone fixation prior to GUS staining, and 3) chloroform treatment prior to acetone fixation and GUS staining. Scale bar 1 mm (n=8-10).

(B) Mean GUS signal intensity values in the SAM and leaf primordia of *bdg1, dcr1-3,* and WT 4-day-old seedlings after control, acetone, and acetone + chloroform treatments. GUS signal was measured using FIJI/ImageJ. Statistical differences were evaluated using two-way ANOVA followed by Tukey's test (n=8-10).

(C) Confocal images showing *PCO1* promoter activity driving GFP expression indicating hypoxia response in the SAMs and leaf primordia of *bdg1 x pPCO1:GUS-GFP* and *pPCO1:GUS-GFP* 3-day-old seedlings after 12 hours of 3.03 kPa oxygen treatment compared to control conditions. The right images show the merged GFP and FM4-64 membrane staining channels, while the left images show only the GFP expression. Scale bar 20 µm (n=7-15).

(D) Mean GFP signal intensity values in the SAMs and leaf primordia of *bdg1 x pPCO1:GUS-GFP* and *pPCO1:GUS-GFP* 3-day-old seedlings (as indicated in the ROI in the images in **C**) were measured using FIJI/ImageJ. Statistical differences were evaluated using two-way ANOVA followed by Tukey's test (n=7-15).

(E) Vibratome sections showing *PCO1* promoter-driven GFP expression indicating hypoxia response in the SAMs and leaf primordia of *bdg1 x pPCO1:GUS-GFP* and *pPCO1:GUS-GFP* 4-week-old plants. Section thickness 120 µm. Scale bar 100 µm (n=7-8).

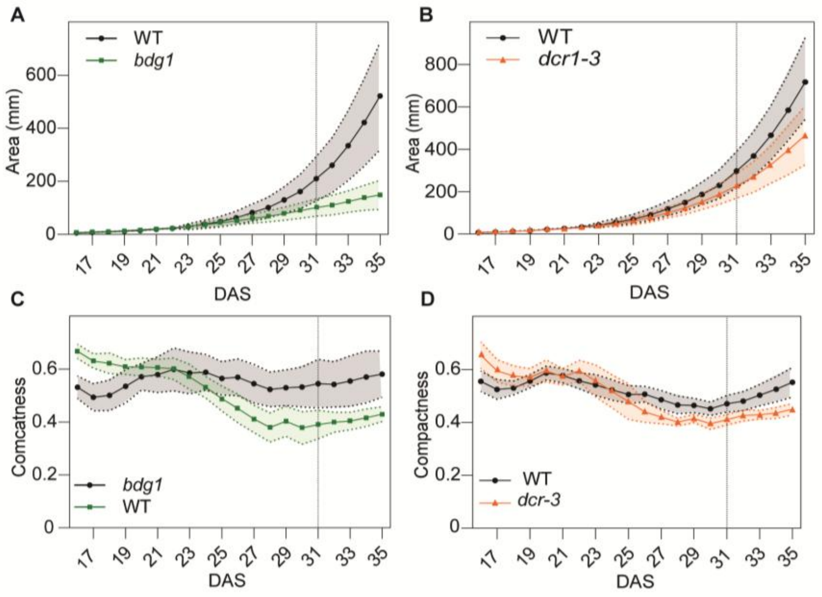

Supplemental figure 8. Phenotypic alterations due to cuticle deficiency.

(A-B) Daily measurements of rosette areas of *bdg1* and *dcr1-3* in comparison to WT plants. Data were collected at the Netherlands Plant Eco-Phenotyping Center (NPEC) using the fully automated plant phenotyping module Helios. Unpaired Student's t-test was used for statistical analysis, with a dotted line indicating comparisons where p < 0,05 started to appear (n=10).

(C-D) Daily measurements of compactness of *bdg1* and *dcr1-3* in comparison to WT plants. Data were collected at the NPEC using plant phenotyping module Helios. Unpaired multiple Student's t-test was used for statistical analysis, with a dotted line indicating comparisons where the p < 0,05 started to appear (n=10).

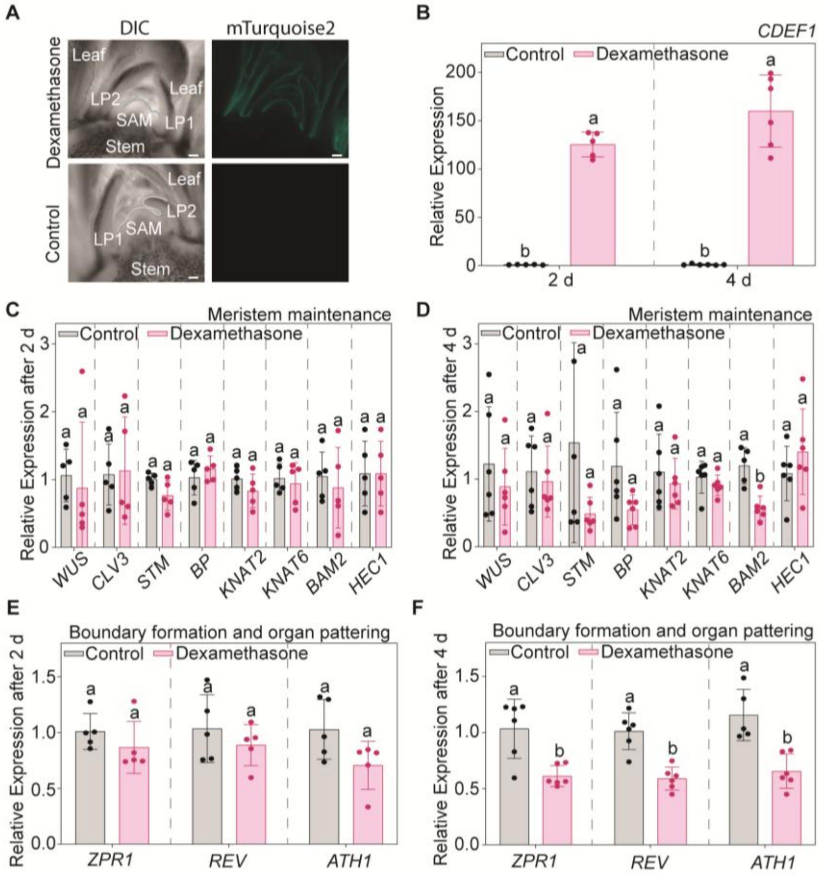

Supplemental figure 9. Gene expression analysis showing meristematic activity after cuticle degradation in the SAM.

(A) mTurquoise visualization in the SAM of 2-week-old *pML1:GR-LHG4 x pOp4:CDEF1* plants treated with dexamethasone. Scale bar 50 µm.

(B) RT-qPCR on 2-week-old shoot apices. Plot shows relative expression of *CDEF1* after 2 and 4 days of dexamethasone treatment vs control. Multiple t-tests with Holm-Sidak correction were used to determine significant differences (n=5-6).

(C-D) RT-qPCR on 2-week-old shoot apices. Plots show relative expression of developmental genes representing meristematic maintenance functions after 2 and 4 days of dexamethasone treatment vs control. Multiple t-tests with Holm-Sidak correction were used to determine significant differences (n=5-6).

(E-F) RT-qPCR on 2-week-old shoot apices. Plots show relative expression of developmental genes representing organ formation, pattering and meristem boundary identity after 2 and 4 days of dexamethasone treatment vs control. Multiple t-tests with Holm-Sidak correction were used to determine significant differences (n=5-6).

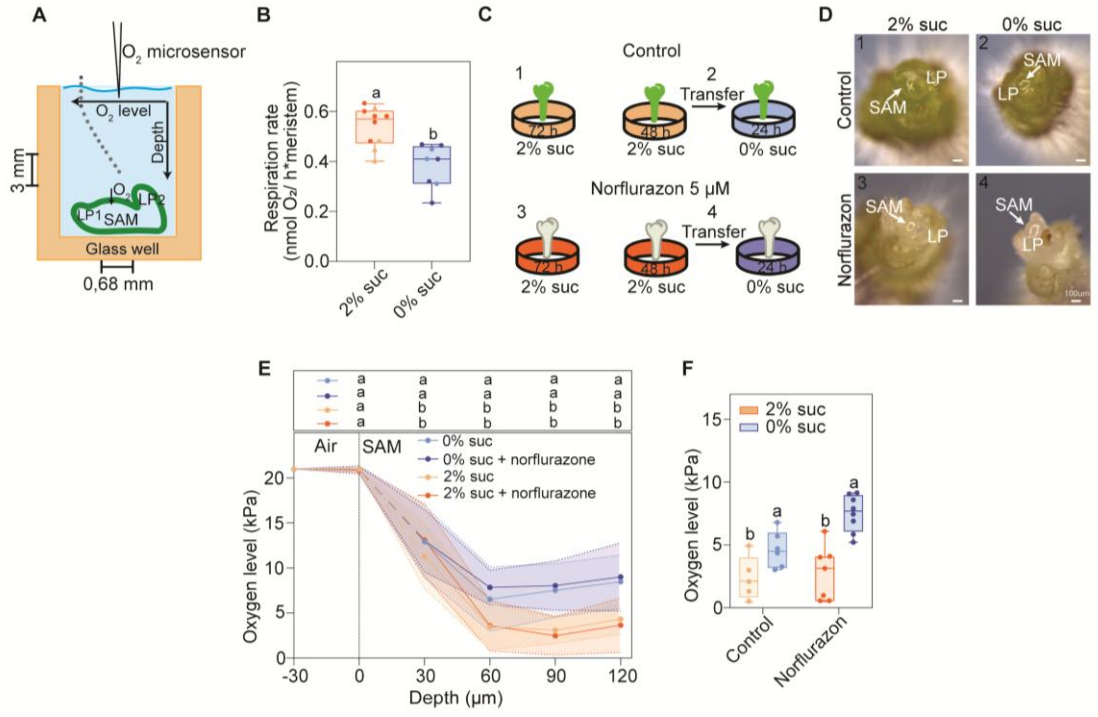

Supplemental figure 10. The effect of resource limitation and removed pigments on oxygen levels in the SAM

(A) Schematic illustration of a single glass rosette well containing the SAM, along with the oxygen profile measured using a Clark-type oxygen microsensor.

(B) Oxygen consumption rates of individual SAMs grown with or without sucrose. Statistical comparison of SAMs grown with and without sucrose was performed using a Student's t-test.

(C) Schematic representation of the experimental setup for the experiments shown in **(D,E,F)**, where dissected tomato SAMs were grown on medium (1) with 2% sucrose (72 h), (2) without sucrose (72 h), (3) with 2% sucrose and 5 µM norflurazon (72h), or (4) transferred from medium with 2% sucrose with 5 µM norflurazon to medium without sucrose with 5 µM norflurazon after 48 h for a 24 h incubation.

(D) Microscopic images of dissected tomato SAM grown: on 2% sucrose medium for 72 h (1), on medium without sucrose for 24 h (2), on 2% sucrose medium with 5 µM norflurazon for 72 h (3) and transferred from medium with 2% sucrose with 5 µM norflurazon to medium without sucrose with 5 µM norflurazon after 48 h with further 24h incubation (4). Scale bar 100 µm.

(E) Clark-type oxygen measurement profiles comparing dissected meristems grown on medium with and without sucrose, with and without norflurazon 5 µM. The vertical stippled line denotes the estimated position of the SAM boundary using microscopy. The dashed segment of the oxygen microprofile indicates the transition of the sensor across this boundary, where oxygen levels are less certain. Statistical differences were evaluated using two-way repeated-measures ANOVA, followed by Tukey's multiple-comparisons test (at each depth), $p < 0.05$, (n=5-8).

(F) Clark-type oxygen measurements at 60 µm depth in tomato SAMs, comparing dissected meristems grown on medium with and without sucrose, with and without norflurazon 5 µM. Statistical differences were evaluated using two-way ANOVA followed by Tukey's test, $p < 0.05$, (n=5-8).

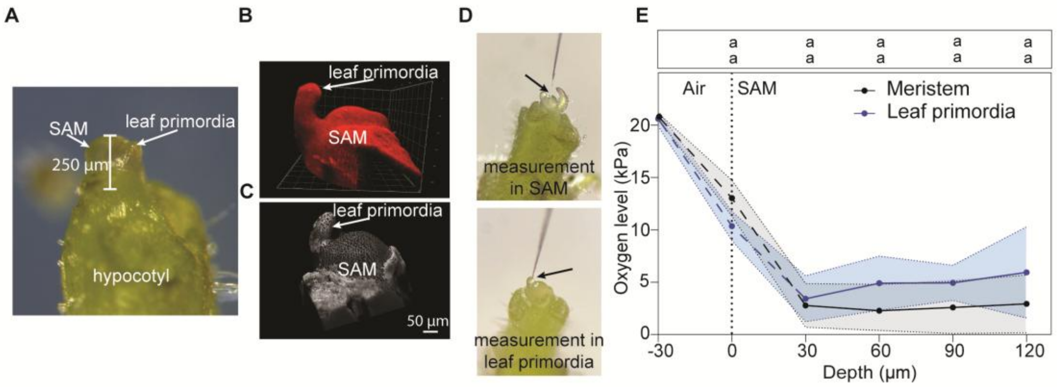

Supplemental figure 11. Volume measurements of the Micro-Tom meristem and oxygen measurements in leaf primordia.

(A) Image of the tomato SAM used for respiration rate measurements with the Unisense nanorespiration setup. The scale bar indicates the region where the meristem was cut from the subapical region.

(B) Confocal maximum intensity projection of a 3D image of a tomato SAM stained with FM4-64. (n=6)

(C) 3D reconstruction generated from the confocal Z-stacks shown in panel **B**.

(D) Images of tomato SAMs showing the oxygen sensor inserted into the meristem (top) and an image where the sensor measured oxygen levels in the leaf primordia (bottom).

(E) Clark-type oxygen measurements showing oxygen profiles in tomato SAM and leaf primordia. The vertical stippled line denotes the estimated position of the SAM boundary using microscopy. The dashed segment of the oxygen microprofile indicates the transition of the sensor across this boundary, where oxygen levels are less certain. Statistical differences were evaluated using two-way repeated-measures ANOVA, followed by Šídák's multiple-comparisons test (at each depth), $p < 0.05$, (n=7).

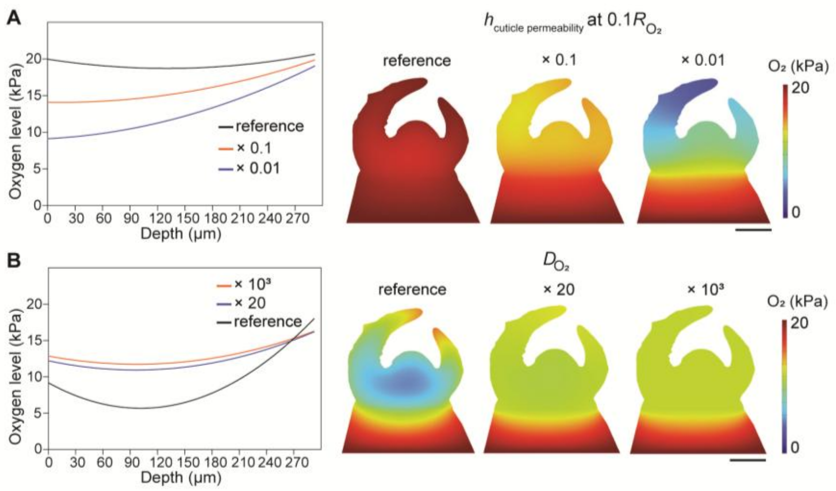

Supplemental figure 12. Modeling oxygen distribution in the apical meristem of tomato.

(A-B) Sensitivity of oxygen profiles to model parameters (showing distribution contours and axial line profiles (scale bar = 100 µm)).

(**A**) Sensitivity to cuticle permeability, at tenfold decreased maximum respiration rate.

(**B**) Sensitivity to diffusivity due to extreme change in porosity.

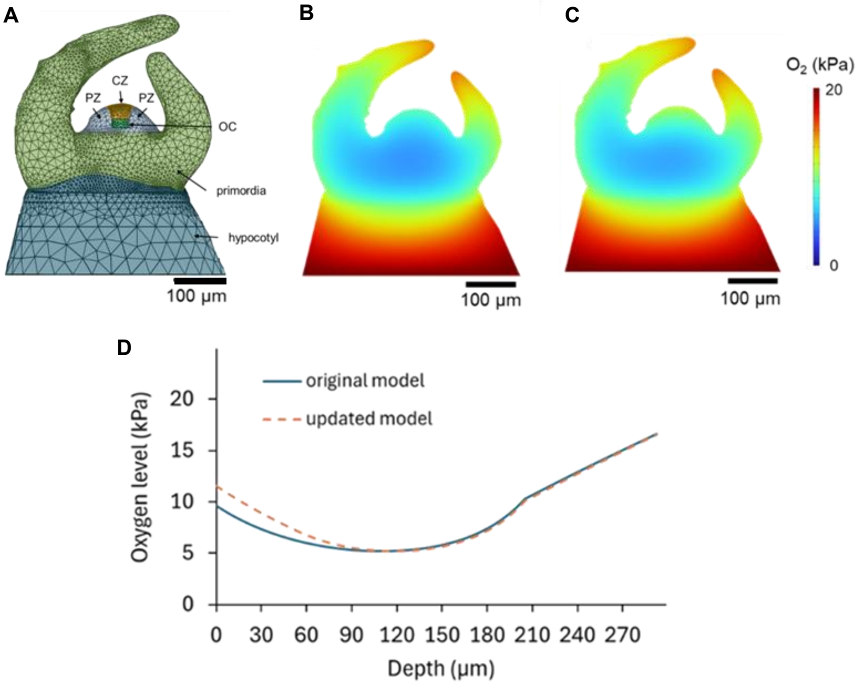

Supplemental figure 13. Oxygen distribution in a heterogeneous shoot apical meristem model.

(**A**) Simulation geometry of the SAM with tissue regions. Following tissues are indicated: CZ (central zone, yellow), OC (organizing centre, dark green), PZ (peripheral zone, light blue), primordia (dark blue) (scale bar = 100 µm). The delineation and volume of these tissues were based on literature, and the volume of each tissue region is given in Supplemental Table 2.

(B-C): oxygen distributions (scale bar = 100 µm). (**B**) Original model. (**C**): updated model.

(**D**) oxygen concentration in the SAM along axial line profiles, comparing the original model (solid blue) and the updated model (dashed orange).

References:

Frost-Christensen, H., Jørgensen, L. B., and Floto, F. (2003). Species specificity of resistance to oxygen diffusion in thin cuticular membranes from amphibious plants. *Plant, Cell and Environment* **26**:561–569.

Kitagawa, M., Balkunde, R., Bui, H., and Jackson, D. (2019). An Aminoacyl tRNA Synthetase, OKI1, Is Required for Proper Shoot Meristem Size in Arabidopsis. *Plant and Cell Physiology* **60**:2597–2608.

MacFarlane, J. J. (1992). Permeability of the cuticle of Vallisneria spiralis to carbon dioxide and oxygen. *Aquatic Botany* **43**:129–135.

Panicucci, G., Iacopino, S., De Meo, E., Perata, P., and Weits, D. A. (2020). An Improved HRPE-Based Transcriptional Output Reporter to Detect Hypoxia and Anoxia in Plant Tissue. *Biosensors (Basel)* **10**:197.

Reddy, G. V., Heisler, M. G., Ehrhardt, D. W., and Meyerowitz, E. M. (2004). Real-time lineage analysis reveals oriented cell divisions associated with morphogenesis at the shoot apex of Arabidopsis thaliana. *Development* **131**:4225–4237.

Schürholz, A.-K., López-Salmerón, V., Li, Z., Forner, J., Wenzl, C., Gaillochet, C., Augustin, S., Barro, A. V., Fuchs, M., Gebert, M., et al. (2018). A Comprehensive Toolkit for Inducible, Cell Type-Specific Gene Expression in Arabidopsis1[CC-BY]. *Plant Physiol* **178**:40–53.

Zabalza, A., Van Dongen, J. T., Froehlich, A., Oliver, S. N., Faix, B., Gupta, K. J., Schmälzlin, E., Igal, M., Orcaray, L., Royuela, M., et al. (2009). Regulation of respiration and fermentation to control the plant internal oxygen concentration. *Plant Physiology* **149**:1087–1098.
